# Novel Hybrid Energy Conversion and Storage Cell with Photovoltaic and Supercapacitor Effects in Ionic Liquid Electrolyte

**DOI:** 10.1038/s41598-018-30707-z

**Published:** 2018-08-15

**Authors:** Saran Kalasina, Nutthaphon Phattharasupakun, Thana Maihom, Vinich Promarak, Taweesak Sudyoadsuk, Jumras Limtrakul, Montree Sawangphruk

**Affiliations:** 1 Department of Chemical and Biomolecular Engineering, School of Energy Science and Engineering, Vidyasirimedhi Institute of Science and Technology, Rayong, 21210 Thailand; 2 Department of Materials Science and Engineering, Vidyasirimedhi Institute of Science and Technology, Rayong, 21210 Thailand

## Abstract

A single hybrid energy conversion and storage (HECS) cell of alpha-cobalt hydroxide (α-Co(OH)_2_) in ionic liquid was fabricated and operated under light illumination. The α-Co(OH)_2_, which is unstable in an aqueous electrolyte (i.e. KOH), is surprisingly stable in 1-butyl-1-methyl-pyrrolidinium dicyanamide ionic liquid. The as-fabricated HECS cell provides 100% coulombic efficiency and 99.99% capacity retention over 2000 cycles. Under a photo-charging condition, the dicyanamide anion of ionic liquid can react with a generated α-Co(OH)_2_^+^ hole at the positive electrode since the HOMO energy level of the anion is close to the valence band of α-Co(OH)_2_. The excited photoelectron will transfer to the current collector and move to the negative electrode. At the negative electrode, the 1-butyl-1-methyl-pyrrolidinium cations of ionic liquid do electrostatically adsorb on the surface and intercalate into the interlayer of active material stabilizing the whole cell. The HECS cell having both energy conversion (photovoltaic effect) and energy storage (supercapacitor) properties may be an ideal device for future renewable energy.

## Introduction

Supercapacitors and batteries are of interest and widely used in many applications including portable electronic devices, electric vehicles, and grid-scale energy storage. Supercapacitors provide high specific power (~500–10,000 W/kg) and long cycle life (>100,000 cycles) but low specific energy^1,2^. Whilst, the batteries offer high specific energy (10–200 Wh/kg) but low specific power^2^. However, both supercapacitors and batteries serve only as energy storage devices and basically provide low energy efficiency over cycling due to heat loss during charging/discharging. They cannot convert energy as thermoelectric and solar cells do^3^. As a result, they cannot harness renewable energy such as solar energy^3,4^. In this work, we introduced a new hybrid energy conversion and storage (HECS) cell of α-Co(OH)_2_ having the photovoltaic effect. In another word, we integrated energy storage and energy conversion systems to a single cell. HECS consists of two processes in a single cell; (i) a semiconductor Co(OH)_2_/liquid electrolyte junction cell generating the photoelectron^5^ and (ii) supercapacitor-based charge storage mechanism process.

**Figure 1:**
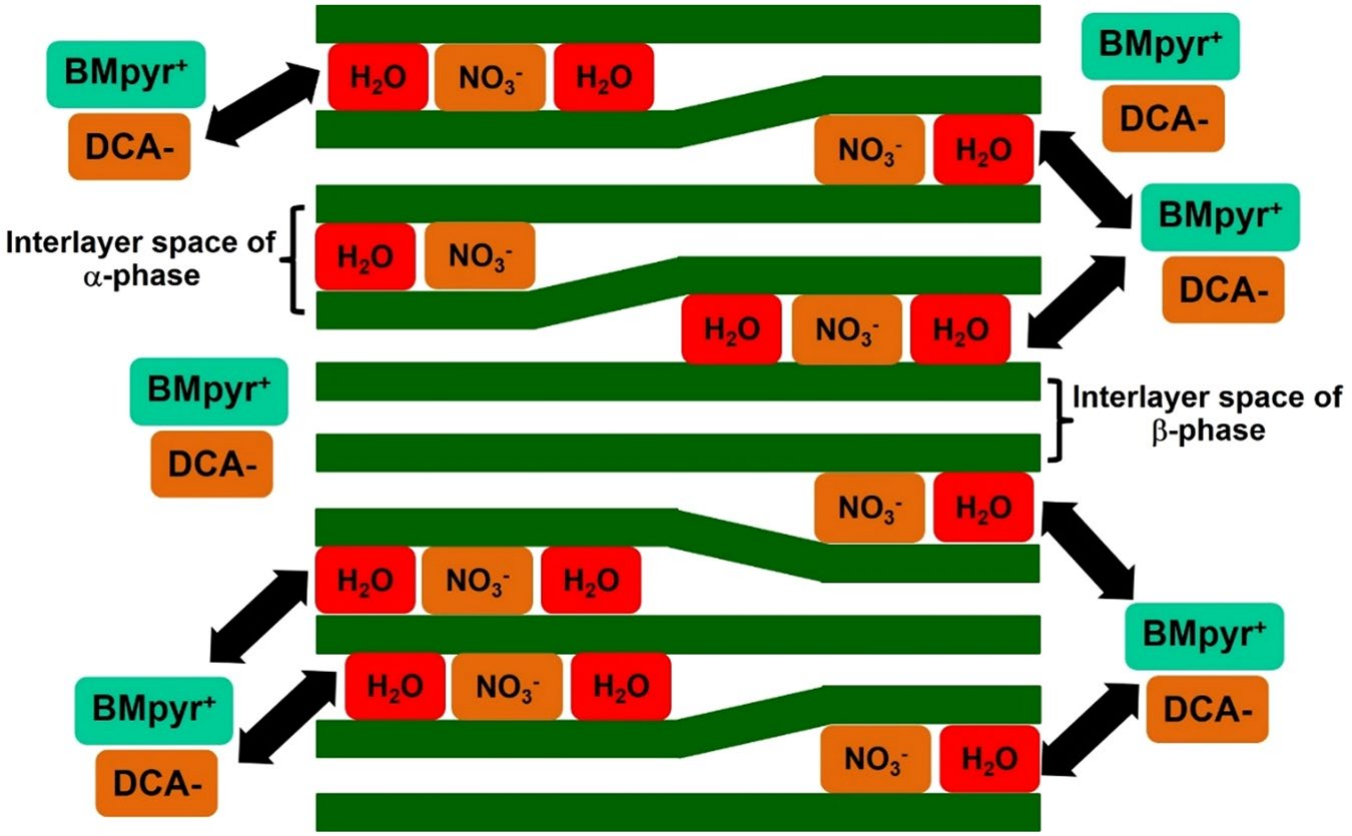
Schematic of the layered Co(OH)_2_; α-phase contains the solvated nitrate anions in its interlayer, whilst the β-phase does not contain any anions in its interlayer. Without polarization, the ionic liquid electrolyte mainly adsorbs at the external surface of Co(OH)_2_.

The layered α-Co(OH)_2_ having an optical band gap energy of *ca*. 2.85 eV can be charged under light illumination having a photoactive HECS behavior. Under light illumination, an electron (e^−^) - hole (h^+^) pair of Co(OH)_2_ can be generated and stored within the material. In addition, the α-Co(OH)_2_ has a two-dimensional nanosheet structure with many advantages i.e., high surface area, porosity, and good electrochemical property. It also provides the interlayer spacing, which can accommodate solvated anions i.e., Cl^−^, NO^3−^, CO_3_^2−^, SO_4_^2−^ in its interlayer^6–9^. The d-spacing value of α-phase Co(OH)_2_ is relatively large (>7 Å) due to the solvated anions between positively charged layers when compared to the β-phase one that does not contain any solvated anions in its interlayer having a narrow d-spacing of ca. 4.6 Å^10,11^. Consequently, the α-Co(OH)_2_ is preferable to other phases for energy storage devices since the d-spacing value of α-phase Co(OH)_2_ is suitable for the intercalation/de-intercalation of the electrolyte as shown in the schematic diagram in Fig. 1.

However, a major drawback of α-Co(OH)_2_ is that its structure is easily collapsed especially once immersed in a basic solution e.g., 1–6 M KOH^12–16^. The hydroxyl ions (OH^−^) can deform the layered structure of α-Co(OH)_2_ via a strong electrostatic interaction between OH^−^ anions and cationic Co(OH)_2_ layer leading to an uncontrolled charge storage mechanism and poor performance^15,16^.

To overcome the phase deformation of α-Co(OH)_2_, herein, the room-temperature 1-butyl-1-methyl-pyrrolidinium dicyanamide ([BMpyr][DCA]) ionic liquid (IL) is for the first time proposed as an alternative electrolyte for the α-Co(OH)_2_-based HECS devices. The electrolyte was selected because it can provide high-stability α-Co(OH)_2_ structure with 99.99% capacity retention and 100% coulombic efficiency. The theoretical calculation along with experimental investigation was used for proposing the charge generation and storage mechanism. In addition, the voltage window of the Co(OH)_2_ HECS cell optimized under dark and blue LED light (470 nm) illumination is rather wide *ca*. 3.5 V implying that it can be practically used in many electronic devices.

## Results and Discussion

### Physicochemical properties

**Figure 2:**
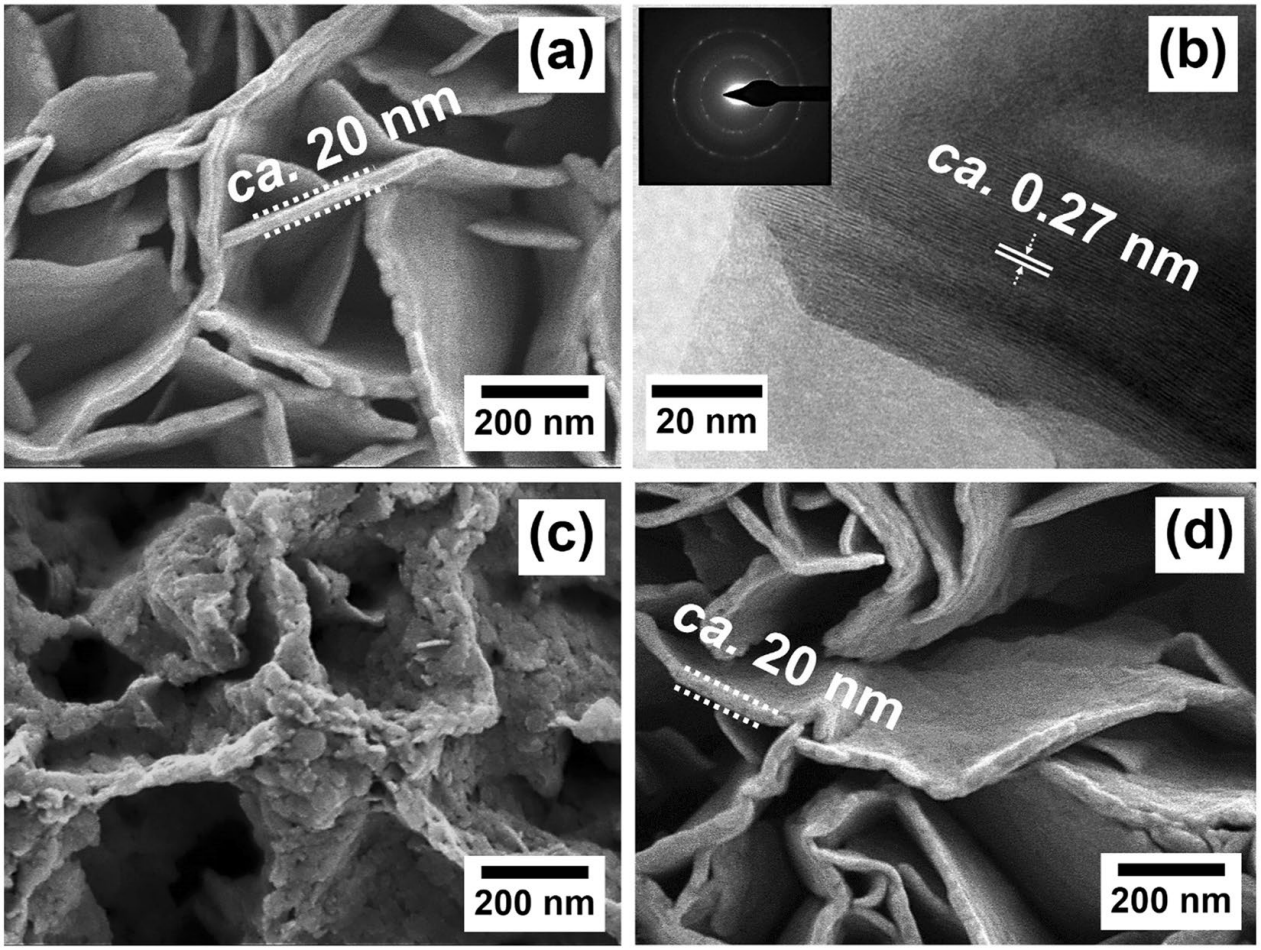
FESEM and HRTEM images of the as-electrodeposited Co(OH)_2_ film (**a,b**), FESEM of the Co(OH)_2_ film after immersed in 6 M KOH for 5 min (**c**) and the as-disassembled Co(OH)_2_ electrode of the ionic liquid-based HECS cell after tested over 2000 cycles (**d**).

The morphology of the as-electrodeposited Co(OH)_2_ film was firstly investigated by FESEM and HRTEM techniques. An FESEM image of Co(OH)_2_ exhibits a nanosheet structure with a thickness of *ca*. 20 nm as shown in Fig. 2a. An HRTEM image of Co(OH)_2_ in Fig. 2b shows the atomic resolution of crystalline α-Co(OH)_2_ structure and their electron diffraction rings. The crystallinity of Co(OH)_2_ film is displayed in an inset image. The diffraction rings can be identified to (101) and (110) lattice planes of the trigonal hydroxide structures (see more details in the supplementary information). A d-spacing value is about 0.27 nm as shown in Fig. 2b, corresponding to the lattice spacing of (101) plane. To show that the α-Co(OH)_2_ was easily deformed after immersed in 6 M KOH for 5 min, an FESEM of the deformed Co(OH)_2_ film is shown in Fig. 2c. This is because OH^−^ can deform the layered structure of α-Co(OH)_2_ via a strong electrostatic interaction between OH^−^ anions and cationic Co(OH)_2_ layer. Also, K^+^ ions in 6 KOH can electrostatically react with anions i.e. NO_3_^−^ inside the interlayer of α-Co(OH)_2_ leading to the phase transformation from α-phase (green color) to β-phase (brown color). On the other hand, the α-Co(OH)_2_ after immersed in [BMpyr][DCA] IL for 5 min shows the same morphology as the as-electrodeposited film. Furthermore, an FESEM image of the as-disassembled Co(OH)_2_ electrode after charged/discharged over 2000 cycles in [BMpyr][DCA] IL has the same morphology as the as-prepared film (Fig. 2d). This suggests that the [BMpyr][DCA] does not deform the α-Co(OH)_2_ structure.

**Figure 3:**
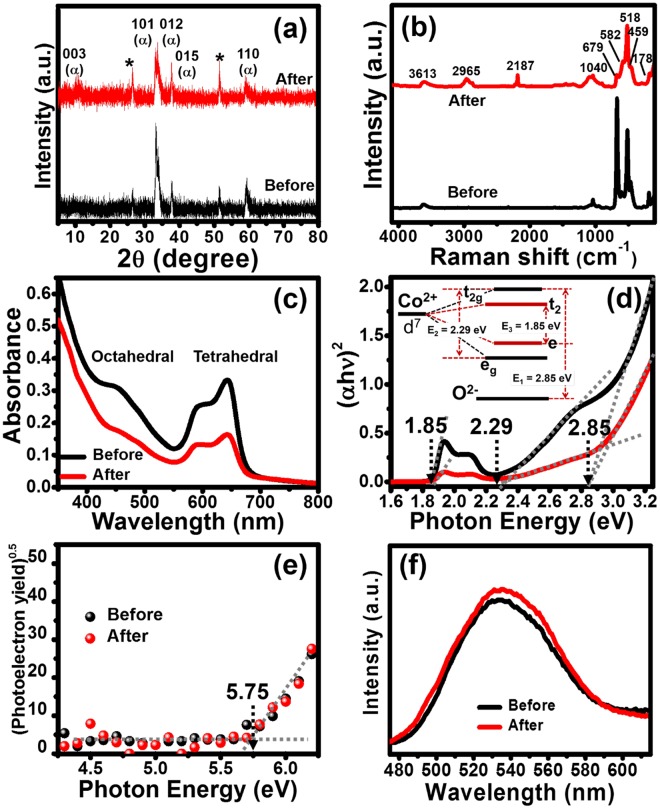
GIXRD patterns (**a**), Raman spectra (**b**), UV-Visible diffuse reflectance spectra (**c**), Tauc plots (**d**), (photoelectron emission yield)^0.5^ as a function of Photon energy (**e**), and Fluorescence excitation spectra (**f**) of Co(OH)_2_ electrodes before (black-line) and after stability test (red line).

To gain better understanding in the structural and electronic properties of the materials, the as-electrodeposited Co(OH)_2_ film and the as-disassembled Co(OH)_2_ electrode after tested for 2000 cycles were investigated with various techniques including Grazing Incidence X-ray Diffraction (GIXRD), Raman spectroscopy, UV-Visible spectroscopy, Photoelectron spectroscopy or Ultraviolet spectrophotometer (UPS), and Fluorescence lifetime spectroscopy (FLS). GIXRD was carried out to investigate the phase transformation of Co(OH)_2_ films as shown in Fig. 3a. The Co(OH)_2_ film before and after stability test displays the similar peaks at 2θ around 10.0^ο^, 32.6^ο^, 33.5^ο^, 37.9^ο^, and 59.9^ο^ corresponding to (003), (101), (012), (015), and (110) planes of α-Co(OH)_2_, respectively. These GIXRD patterns are in good agreement with the diffraction rings observed in HRTEM and their selected area electron diffraction (SAED) data (see more details in the supplementary information). Note that the FTO substrate peaks can be observed in the measurement as marked with the * symbol. The purity of the electrodes before and after tested is also rather high (see FESEM/EDX in Fig. S2). In addition, the powder of the as-scrapped Co(OH)_2_ film after the electrodeposition was investigated by XRD. The d-spacing of both α-Co(OH)_2_ and β-Co(OH)_2_ calculated is shown in Fig. S1c. The d-spacing of α-Co(OH**)**_2_ calculated from the 003 plane (2θ = 10.1^ο^) is *ca*. 8.8 Å. Whilst, the d-spacing of β-Co(OH**)**_2_ calculated from the 001 plane (2θ = 19.9^ο^) is *ca*. 4.5 Å. The ionic size of dicyanamide anion (DCA^−^) of IL estimated from the geometry optimization is *ca*. 4.4 Å and that of 1-butyl-1-methyl-pyrrolidinium cation (BMpyr^+^) is *ca*. 8.0 Å. The results confirm that the IL can diffuse into the interlayer of α-Co(OH)_2_ structure leading to the intercalation/de-intercalation chemistry.

The Raman spectra of Co(OH)_2_ before and after electrochemically tested are shown in Fig. 3b. The as-electrodeposited Co(OH)_2_ film displays two main peaks at around 500 and 670 cm^−1^ which can be ascribed to the vibration mode of Co-O bonding and the scissor mode of nitrate ions (NO_3_^−^)^17–19^. The vibration modes of nitrate ions and hydroxyl groups (OH^−^) of α-Co(OH)_2_ also occur at ~1040 and 3600 cm^−1^, respectively^20–22^. In addition, the peaks at 467 and 186 cm^−1^ can be assigned to the O-Co-O bending mode and lattice vibration, respectively^17,18^. After the stability test, the as-disassembled Co(OH)_2_ electrode shows a major peak at *ca*. 500 cm^−1^ confirming that the Co-O bonding remains in the Co(OH)_2_ electrode after tested while the intensity of the peak at *ca*. 670 cm^−1^ is reduced due to the IL adsorption (see Fig. S12). The residual NO_3_^−^ and OH^−^ peaks (~1040 and 3600 cm^−1^) clearly suggest that the Co(OH)_2_ electrode after charged/discharged for 2000 cycles remains α-phase. In addition, some peaks of the adsorbed [BMpyr][DCA] IL can also be observed at ~2965 and ~2187 cm^−1^ (see Raman and FTIR spectra of [BMpyr][DCA] IL and Co(OH)_2_-IL in Fig. S3).

As the optical properties of cobalt compounds e.g., α-Co(OH)_2_, β-Co(OH)_2_, and Co_3_O_4_ are clearly different, they can be beneficial for investigating the phase transformation of α-Co(OH)_2_. The Co(OH)_2_ electrodes before and after tested were investigated by UV-Visible diffuse reflectance spectroscopy and their absorption spectra are shown in Fig. 3c. They exhibit the similar absorption spectra at wavelengths of *ca*. 450, 590, and 650 nm. The absorption band at *ca*. 450 nm is a result of octahedral of Co^2+^ whilst the absorption peaks at *ca*. 590 and 650 nm can be referred to the tetrahedral of Co^2+^ ^23–25^. The Co(OH)_2_ consists of both octahedral and tetrahedral sites of Co^2+^ leading to an overlapping of energy level in the 3d orbital of Co^2+^. The energy states of the tetrahedral site (e and t_2_) with a smaller gap overlap with the energy states of the octahedral site (e_g_ and t_2g_) as shown in the inset image of Fig. 3d. With these peak positions in the UV-Visible spectrum of α-Co(OH)_2_, three energy gaps can be estimated at 1.85, 2.29, and 2.85 eV, respectively as shown in Fig. 3d. Each energy gap relates with the different transition states as shown in the inset. The first energy gap (E_1_) is *ca*. 2.85 eV, corresponding to the transition state of 2p (O^2−^) → t_2g_ 3d (Co^2+^), whilst the second energy gap (E_2_) is *ca*. 2.29 eV, corresponding to the transition state of e_g_ 3d (octahedral, Co^2+^) → t_2g_ 3d (octahedral, Co^2+^) and the third energy gap (E_3_) is *ca*. 1.85 eV, corresponding to the transition state of e 3d (tetrahedral, Co^2+^) → t_2_ 3d (tetrahedral, Co^2+^). In addition, the optical energy gap was also calculated using a Tauc formula (see more details in the supplementary information)^26^. The relationships between (αhν)^2^ and photon energy were plotted, and the optical energy gaps can be found on the X-intercept of the straight line as shown in Fig. 3d. The Co(OH)_2_ samples before and after tested show the similar optical energy gap of 2.85 eV suggesting that it is rather stable after tested over 2000 cycles.

UPS and FLS were measured to confirm that α-Co(OH)_2_ is a photoactive material, which can be excited with light. The ionization energy of both the as-electrodeposited and the as-tested Co(OH)_2_ films was measured to estimate the minimum amount of photon energy for electron excitation corresponding to the valence band (VB) edge (see more details in the supplementary information). The VB edge can be obtained from the relationship between the square root of photoelectron emission yield and photon energy as shown in Fig. 3e. The VB of the as-electrodeposited α-Co(OH)_2_ is about -5.75 eV, which is the same value as that of the as-disassembled Co(OH)_2_ electrode as shown in Fig. 3e. The fluorescence emission spectra of Co(OH)_2_ films before and after tested were also measured by FLS with the same excitation energy of 430 nm. The fluorescence emission spectra of both Co(OH)_2_ exhibit a unique single peak at around 535 nm (green color), which is the characteristic of α-Co(OH)_2_ film as shown in Fig. 3f.

### Photoelectrochemical evaluation

**Figure 4:**
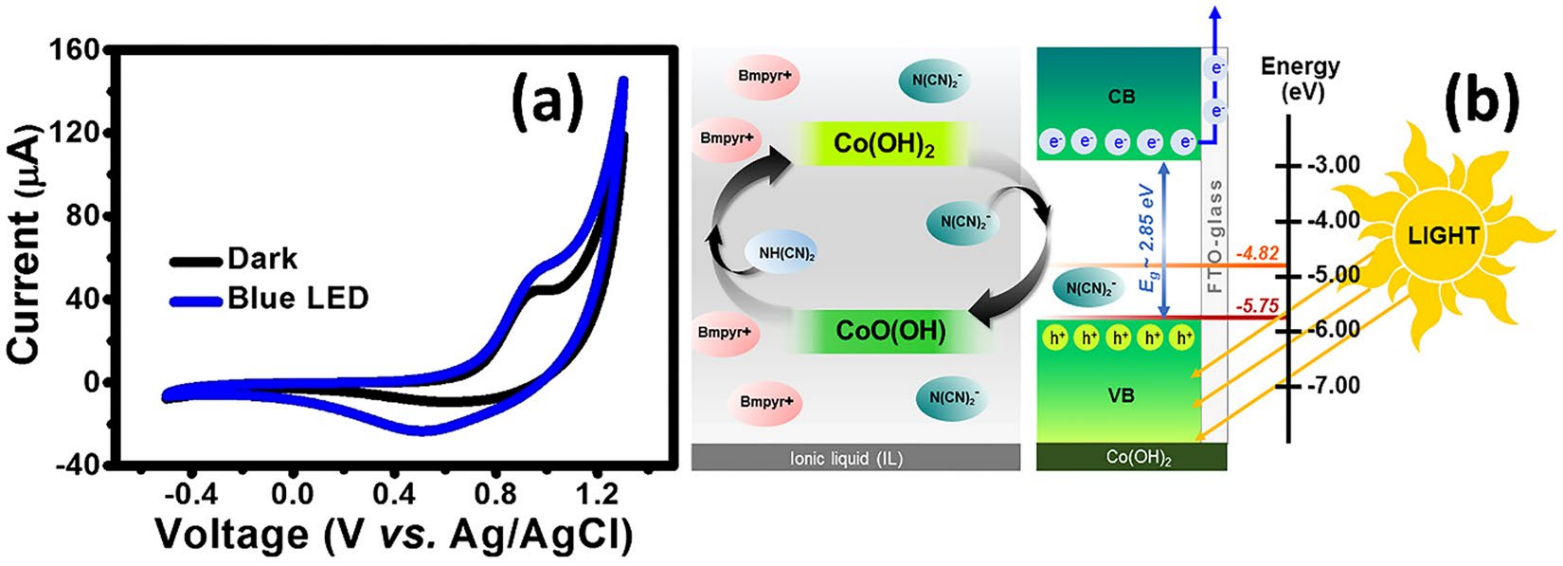
CVs of the half-cell Co(OH)_2_ electrode in IL electrolyte carried out under dark condition (black line) and blue LED light illumination (blue line) (**a**) and the schematic of the electron transfer and charge storage mechanisms (**b**).

For the electrochemical measurement, the half-cell α-Co(OH)_2_ electrode was firstly investigated by cyclic voltammetry (CV) in [BMpyr][DCA] IL. CV was evaluated in a potential range of -0.5–1.3 V at a scan rate of 10 mV/s under dark condition and blue LED light illumination as shown in Fig. 4a. The CV curve under blue LED light illumination exhibits a pair of redox peaks indicating to the reversible redox reaction of α-Co(OH)_2_ in [BMpyr][DCA] IL electrolyte. This supports the proposed schematic as shown in Fig. 1 and relates with the calculated d-spacing from XRD. The DCA^−^ (*ca*. 4.4 Å in size) can diffuse into the interlayer of α-Co(OH)_2_ structure having the d-spacing of *ca*. 8.8 Å during the anodic scan (-0.5 to 1.3 V vs. Ag/AgCl). Note, the commercial screen-printed Ag/AgCl was used as the reference electrode. Also, the BMpyr^+^ (*ca*. 8.0 Å in size) can intercalate into the interlayer of α-Co(OH)_2_ structure during the cathodic scan (1.3 to -0.5 V vs. Ag/AgCl). Interestingly, the larger current density of the half-cell of α-Co(OH)_2_ electrode under light illumination can be clearly observed when compared to that under the dark condition. The increase in the current response under light illumination can be ascribed to the photo-charging redox reaction mechanism of Co(OH)_2_ material and DCA^−^ as proposed by the following reactions (1 and 2) and the schematic in Fig. 4b. The α-Co(OH)_2_ can initially be excited with a blue LED light generating the Co(OH)_2_^+^ hole and electron (e^−^) pairs as the following reaction (1). Subsequently, N(CN)_2_^−^ (DCA^−^) having higher energy level (-4.82 eV) than Co(OH)_2_^+^ hole (-5.75 eV) can react with Co(OH)_2_^+^ hole providing CoO(OH) and NH(CN)_2_ products according to the reaction (2). During the charging process, the Co(OH)_2_ gets oxidized to CoO(OH) and reduced back to Co(OH)_2_ at the discharging process as shown in the overall reversible redox reaction (3).

1 $${\boldsymbol{Co}}{({\boldsymbol{OH}})}_{2}\mathop{\longleftrightarrow }\limits^{{\boldsymbol{hv}}}{\boldsymbol{Co}}{({\boldsymbol{OH}})}_{2}^{+}+{{\boldsymbol{e}}}^{-}$$

2 $${\boldsymbol{Co}}{({\boldsymbol{OH}})}_{2}^{+}+{\boldsymbol{N}}{({\boldsymbol{CN}})}_{2}^{-}\leftrightarrow {\boldsymbol{CoO}}({\boldsymbol{OH}})+{\boldsymbol{NH}}{({\boldsymbol{CN}})}_{2}$$

3 $${\boldsymbol{Co}}{({\boldsymbol{OH}})}_{2}+{\boldsymbol{N}}{({\boldsymbol{CN}})}_{2}^{-}\mathop{\longleftrightarrow }\limits^{{\boldsymbol{hv}}}{\boldsymbol{CoO}}({\boldsymbol{OH}})+{\boldsymbol{NH}}{({\boldsymbol{CN}})}_{2}+{{\boldsymbol{e}}}^{-}$$

**Figure 5:**
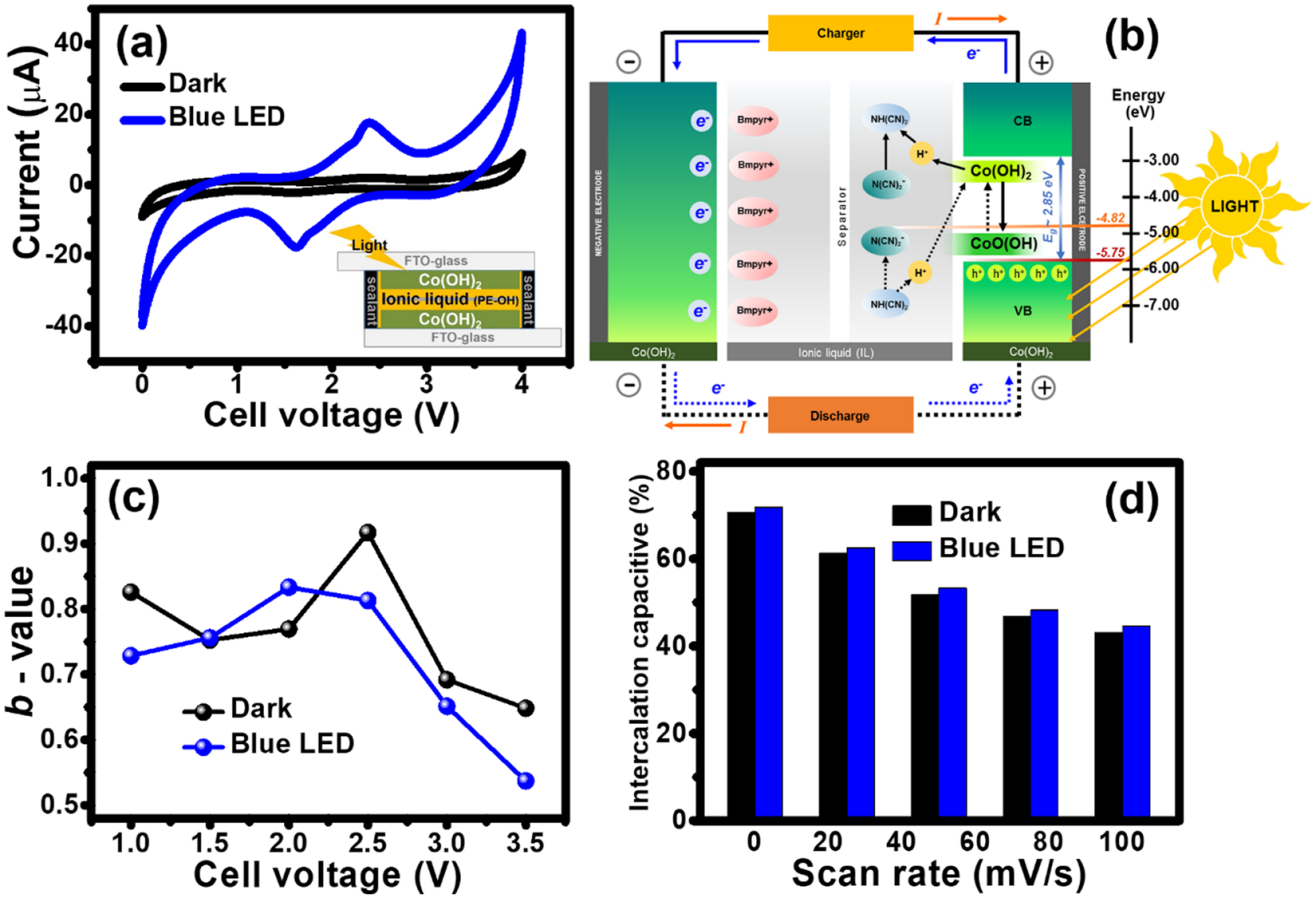
CVs of the symmetric Co(OH)_2_ HECS in IL electrolyte measured under dark condition (black line) and blue LED light illumination (blue line) (**a**), the schematic of the charge storage mechanism of the symmetric Co(OH)_2_ HECS in IL electrolyte under light illumination (**b**), the calculated *b*-values as a function of applied potentials (**c**), and the bar charts of the intercalation capacitive as a function of scan rates (**d**).

For the symmetric α-Co(OH)_2_ HECS cell, it was fabricated using [BMpyr][DCA] IL electrolyte and hydrolyzed polyethylene separator. The charge storage performance of the cell was investigated by cyclic voltammetry (CV) and galvanostatic charge-discharge (GCD). CV was firstly evaluated in a wide potential range of 0–4.0 V at the scan rates of 10, 25, 50, 75, and 100 mV/s to see the clear reversible redox reaction (see all CV curves in the supplementary information). The CV curves of the Co(OH)_2_ HECS cells were measured under dark condition and blue LED light illumination as shown in Fig. 5a. The CV curve under blue LED light illumination displays the symmetric redox peaks indicating the reversible redox reaction of α-Co(OH)_2_ in [BMpyr][DCA] IL electrolyte. To gain better understanding on the photo-charging process of the Co(OH)_2_ HECS cell, it is proposed as shown in Fig. 5b. Under light illumination, the photo-charging mechanism can generate electron-hole in Co(OH)_2_ positive electrode, whilst the anions (DCA^−^) diffuse towards the positive electrode and cations (BMpyr^+^) diffuse to the negative electrode. The free electrons from the positive electrode then transfer to the negative electrode that will adsorb the counter cations during the charging process to stabilize the cell. For the discharging process, the cations will desorb from the surface of the negative electrode and recombine with their anion pairs and become neutral in the bulk electrolyte within the polymer separator. Note that this proposed mechanism is corresponding to that of Ni-MH battery (Fig. S13**)**. In addition, the higher current density of the HECS under light illumination can be clearly observed at all applied scan rates when compared to the dark condition (see all CVs in the supplementary information). The increase of the current under light illumination can be ascribed to the photocurrent generated from the photo-charging mechanism of Co(OH)_2_ material as proposed in the reactions (1–3).

In general, the CV curve is composed of two charge storage mechanisms contributing from diffusion-controlled intercalation process (battery-type behavior) and surface capacitive process (EDLC and pseudocapacitive). To separate these two contributions, the power law was applied at various scan rates (*i* = *aυ*^b^)^27–29^. The *b*-value can be obtained from the slope of the relationship between log (*i*) and log (υ) (see more details in the supplementary information). At the *b*-value of 0.5, the currents are proportional to the square root of scan rates, indicating the diffusion-controlled intercalation process or battery-type behavior. As the *b*-value reaches 1.0, it can be referred to the surface capacitive effect, including EDLC and surface redox^30^. To identify these contributions, the *b*-value was calculated from the CVs and plotted as a function of various potentials as shown in Fig. 5c. The calculated *b*-value lies in the range of 0.5–1.0 indicating the combination of both surface capacitive effect and diffusion-controlled intercalation process. At the potentials of 1.0, 1.5, 2.0, and 2.5 V, the calculated *b*-values are more than 0.7 and then decrease when the potentials increase to 3.0 and 3.5 V. Both cells under dark condition and blue light illumination display the similar trend. At the window potential of 3.5 V, the HECS under blue LED illumination provides lower *b*-value than that under dark condition due to the photo-charging redox mechanism of Co(OH)_2_.

From the equation (S4) in the supplementary information, the CV data can be converted into the fraction between surface capacitive effects and diffusion-controlled intercalation processes (see more details in the supplementary information). The bar chart of the intercalation capacitive percentages as a function of scan rates is shown in Fig. 5d. Increasing scan rates significantly affects the intercalation capacitive of the HECS devices for which most ions will not be able to diffuse into Co(OH)_2_ layers at fast scan rates due to diffusion limit. However, the cell under blue LED illumination displays higher values resulting from the photo-charging effect following the reactions (1–3) above. This suggests that Co(OH)_2_ exhibits a battery-type charge storage behavior under dark and blue LED light illumination.

**Figure 6:**
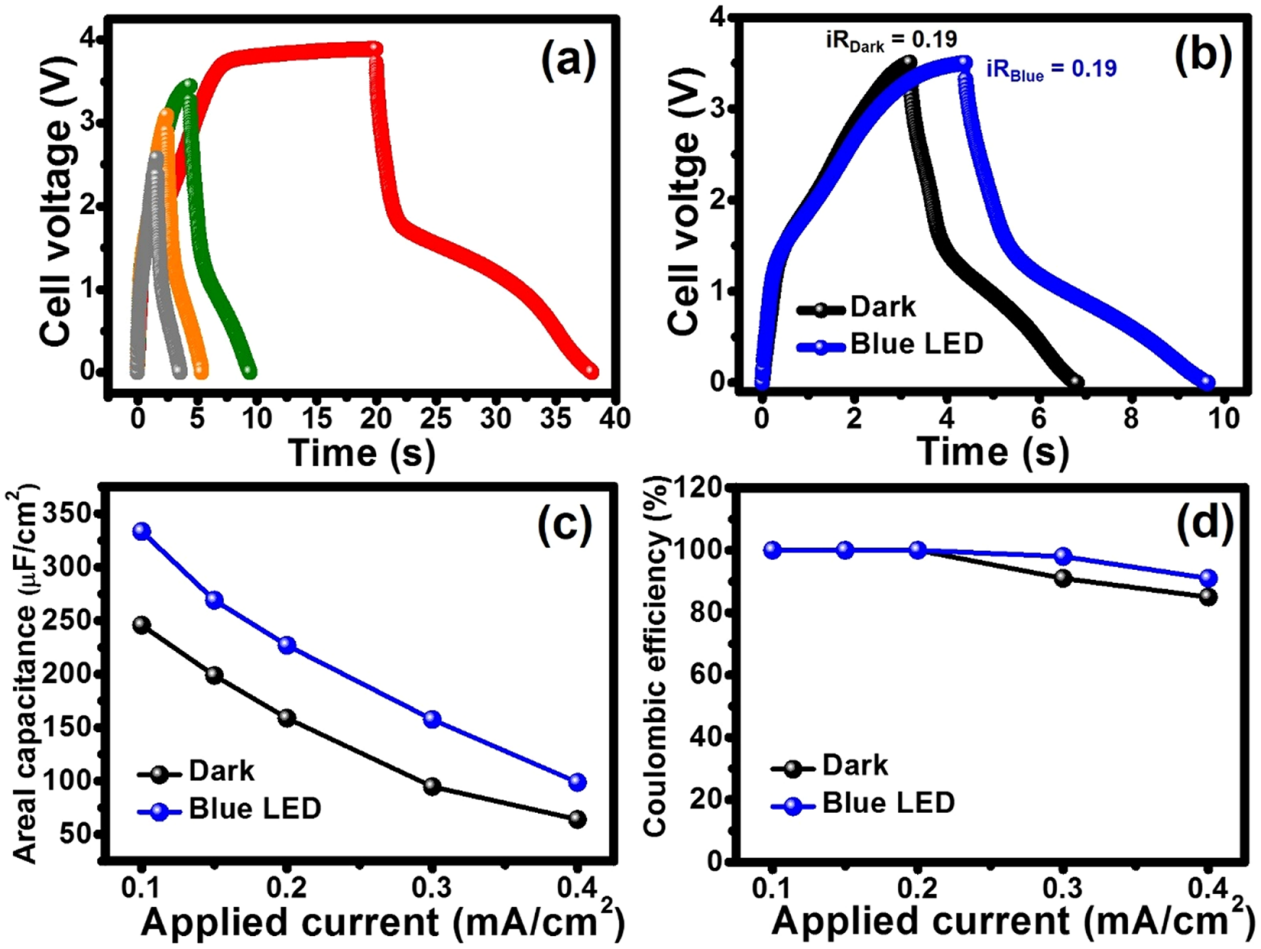
GCDs at 0.1 mA/cm^2^ with different working voltages under blue LED light illumination (**a**), GCDs at 0.1 mA/cm^2^ at a potential window of 3.5 V (**b**), and the areal capacitances as a function of the applied currents (**c**), Coulombic efficiency as a function of the applied currents. GCD was measured under dark condition (black line) and blue LED light illumination (blue line).

To further evaluate the electrochemical performance of the HECS cell, the GCD was carried out at various voltage windows of 2.5, 3.0, 3.5, and 4.0 V under blue LED light illumination to avoid overcharging effect and huge *iR* drop as shown in Fig. 6a. At the voltage windows of 2.5, 3.0, and 3.5 V, the Co(OH)_2_ HECS cell with IL provides high coulombic efficiency up to 100% without any signs of electrolyte degradation or overcharging (see their coulombic efficiency values in the supplementary information). Whilst, at a voltage window of 4.0 V, the cell displays the unsymmetrical shape of GCD profile with an overcharging characteristic suggesting that the optimum voltage window should be determined at *ca*. 3.5 V. Note that this is in good agreement with the *b*-value that closes to 0.5 at 3.5 V. In addition, the GCDs under dark condition were also measured at different voltages (see more details in the supplementary information) showing the same trends as the cell under blue LED light illumination.

The GCD was further investigated by applying various current densities (0.1–0.4 mA/cm^2^) at a voltage window of 3.5 V under dark condition and blue LED light illumination (see more GCD results in the supplementary information). The GCD curves at 0.1 mA/cm^2^ are shown in Fig. 6b. The GCD curve under illumination condition exhibits longer charge-discharge time than that under dark condition, indicating that blue LED light significantly contributes to the charge storage mechanisms as proposed in reactions (1–3). The calculated areal capacitances as a function of applied current densities are shown in Fig. 6c. At 0.1 mA/cm^2^, the Co(OH)_2_ HECS cell under the blue LED light illumination exhibits an areal capacitance of 333 μF/cm^2^, which is 1.36-fold higher than that under dark condition (245 μF/cm^2^). The areal capacitances at the applied currents of 0.15, 0.2, 0.3, 0.4 mA/cm^2^ under the blue light illumination are 269, 227, 157, and 98 μF/cm^2^, respectively. Whilst, under dark condition they are 198, 158, 94 and 64 μF/cm^2^, respectively. In addition, the Coulombic efficiencies as a function of the applied currents are also shown in Fig. 6d. At the applied currents of 0.1, 0.15, and 0.2 mA/cm^2^ under dark and light conditions, both cells exhibit the coulombic efficiency of ca. 100% and then decrease at the higher applied currents of 0.3 and 0.4 mA/cm^2^.

**Figure 7:**
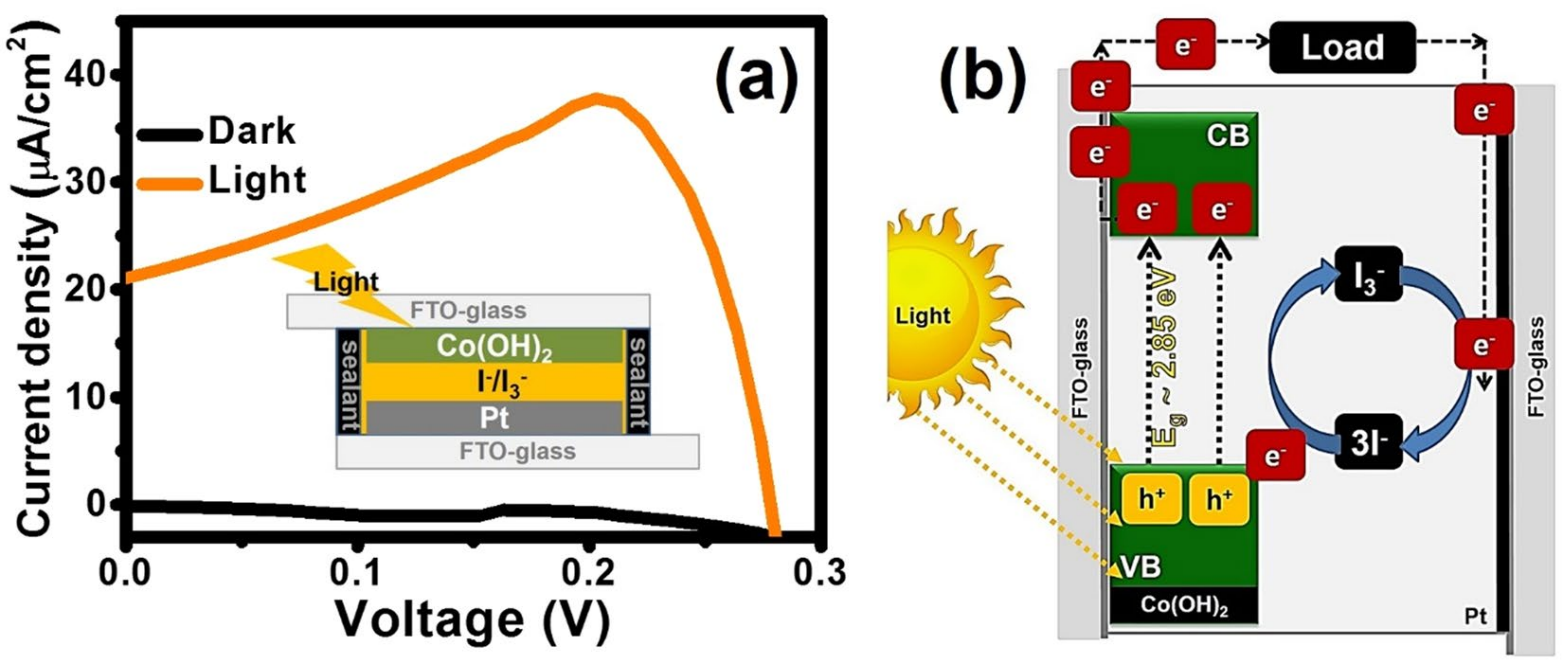
*I-V* curves of Co(OH)_2_ film used as the photoelectrode of the semiconductor/liquid junction photovoltaic cell measured under dark condition (black line) and light illumination (orange line) (**a**) and the schematic of Co(OH)_2_ photoelectrode in the photovoltaic cell (**b**).

To gain better understanding about the photovoltaic effect and electron transfer of Co(OH)_2_ photoelectrode, a semiconductor/liquid junction photovoltaic cell of α-Co(OH)_2_ using iodide/triiodide (I^−^/I_3_^−^) redox mediator as the electrolyte and Pt film as the counter electrode (CE) was fabricated^5^. The cell was investigated by the current-voltage characterization (I-V curve) under dark and light illumination conditions as shown in Fig. 7a. Under dark condition, the current density of the cell closes to zero in contrast to the cell under light illumination having photocurrent for which the current density (*J*_*sc*_) of the cell under light illumination is 10.38 μA/cm^2^ with the open circuit voltage (*V*_*oc*_) of 0.27 V, the Fill Factor (*FF*) of 1.26, and the efficiency of 3.49 × 10^−3^ %. This supports that the α-Co(OH)_2_ material can serve as the photoelectrode with the photovoltaic effect. The photoelectron transfer in the cell can be explained by a schematic in Fig. 7b. When the α-Co(OH)_2_ is excited under light, the electrons from VB move to CB and then flow from Co(OH)_2_ electrode to the load and CE, respectively. The electron produced from the reversible redox reaction of I^−^/I_3_^−^ redox provides electron back to the hole of Co(OH)_2_ to stabilize the whole cell. As a result, it can prove our new designed HECS cell of Co OH)_2_.

**Figure 8:**
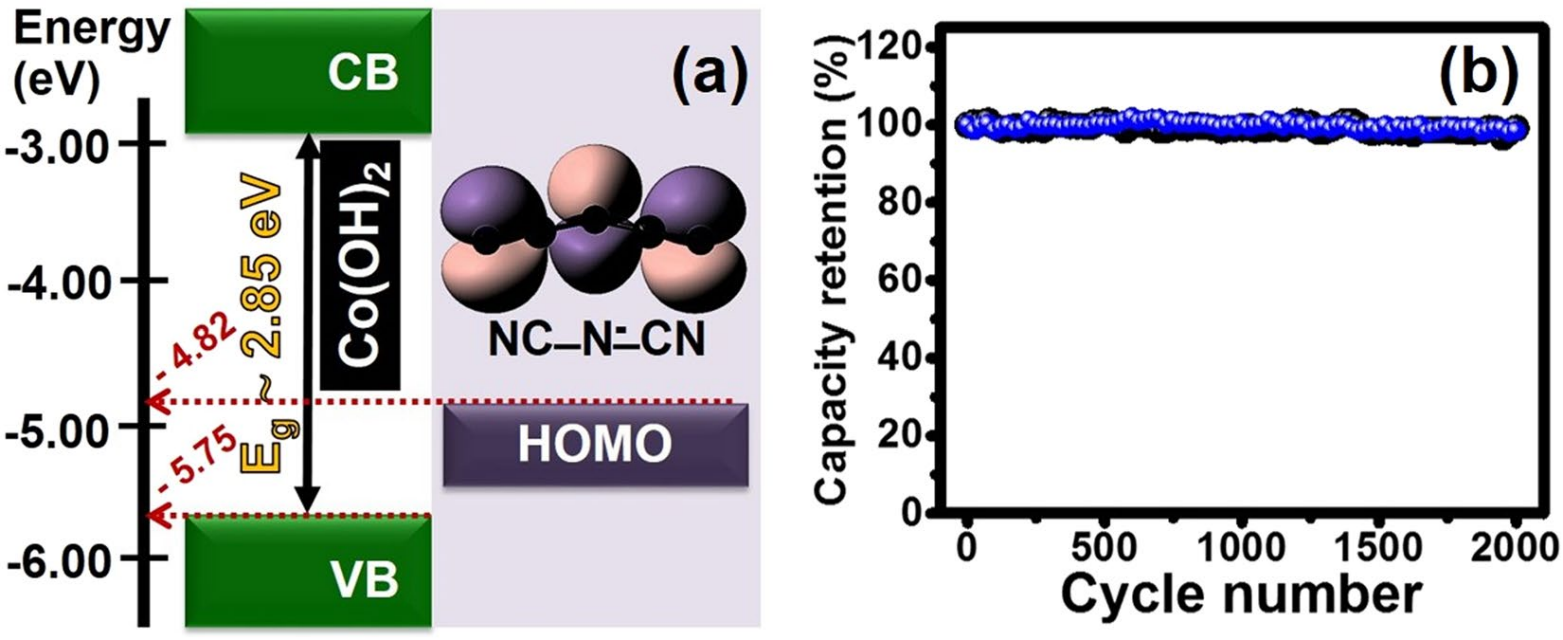
The relationship of the energy level of Co(OH)_2_ with dicyanamide anion (DCA^−^) of [BMpyr][DCA] IL (**a**) and the capacity retention over 2000 cycles (**b**) under dark condition (black line) and blue LED light illumination (blue line).

To gain better understanding on the photo-charging mechanisms of Co(OH)_2_ in the [BMpyr][DCA] IL as proposed in reactions (1–3), the relationship of energy level between Co(OH)_2_ and dicyanamide anion (DCA^−^) molecule or (N(CN)_2_^−^) of [BMpyr][DCA] IL was theoretically investigated by an ab-initio calculation as shown in Fig. 8a. The ground-state geometry optimizations were carried out using the MP2 method and the aug-cc-pVTZ basis set (see more details in the supplementary information). The highest occupied molecular orbital (HOMO) was found to be delocalized predominantly on all atoms of DCA with π*-orbital. The HOMO energy level was calculated to be −4.82 eV. Note, the valence band of Co(OH)_2_ measured by the UPS is about −5.75 eV. The calculated HOMO of DCA is slightly higher than the valence band of Co(OH)_2_ suggesting that the DCA^−^ anion can easily react with the hole (Co(OH)_2_^+^) as proposed in the reaction (2). Note, other anion compounds were also theoretically investigated but it turned out that the DCA is the optimum one (see Table S3).

Finally, the cycling performance of the Co(OH)_2_ HECS cell in the IL electrolyte was tested under dark and blue LED light illumination for 2000 cycles as shown in Fig. 8b. The cells exhibit an excellent capacity retention (99.99%). In addition, the electrode also maintains a conventional green color after tested (see Fig. S8) indicating that the α-Co(OH)_2_ is stable in the IL electrolyte over long cycling. Unlike the cell in the KOH electrolyte, the color of the as-tested electrode is turned from green (α-phase) to brown (β-phase) and black (Co_3_O_4_) films, respectively^16^. For long-term cycling stability (up to 4000 cycles), the capacity retention of the Co(OH)_2_ HECS cell remains at *ca*. 90%. Note, the decrease in stability can be ascribed to the strong redox reaction causing a slight change in the morphology and phase of α-Co(OH)_2_^31,32^.

## Conclusions

In summary, we introduced a new hybrid energy conversion and storage device (HECS) of layered α-Co(OH)_2_. This semiconductor material having an optical band gap of 2.85 can absorb the blue light generating the hole-electron pair via a photoelectric effect. However, it is not stable at all especially in aqueous electrolytes (e.g. KOH), which is rather different from its family material layered α-Ni(OH)_2_ widely used in NiCd and NiMH batteries having rather high stability in 6 M KOH. In this work, we used [BMpyr][DCA] IL as the replacement of KOH electrolyte with an objective to prevent the phase transformation of layered α-Co(OH)_2_ to β-phase and Co_3_O_4_. The Co(OH)_2_ HECS devices using IL were electrochemically tested under dark and blue LED light illumination to confirm the photo-charging behavior. The charge storage mechanism was investigated by means of FLS, UPS, and DFT calculation. The anion (DCA^−^) of IL was found to reversibly react with the Co(OH)_2_^+^ hole after excited with light producing cobalt oxyhydroxide (CoO(OH)) and NH(CN)_2_. This is because the HOMO of DCA^−^ anion is close to the valence band of Co(OH)_2_. After tested for 2000 cycles, the Co(OH)_2_ exhibits the same physical and chemical properties as the as-electrodeposited Co(OH)_2_ without any deformation. This integration between energy storage and energy conversion system under IL electrolyte will provide an opportunity to expand the horizons of energy technology and open new windows in this research area.

## Experimental Section

### Electrodeposition of α-Co(OH)_2_ on FTO glass substrate

Firstly, FTO glasses were cleaned with detergent, DI water, absolute ethanol, and acetone, respectively. The cleaned 1 cm × 1 cm FTO glasses were used as current collector or substrate for the electrodeposition of α-Co(OH)_2_ film. The counter and reference electrodes are platinum wire and Ag/AgCl (3 M KCl), respectively. An aqueous solution of 0.1 M Co(NO_3_)_2_.6H_2_O (analytical grad, UNIVAR) in 0.5 M NaNO_3_ (analytical grad, UNIVAR) was used as an electrodeposition solution. Three electrodes were immersed in the solution and the α-Co(OH)_2_ film was obtained by applying -1.0 V *vs*. Ag/AgCl for 1 min (for half-cell measurement) and 5 min (for symmetric full-cell measurement) via a chronoamperometry method using a Metrohm AUTOLAB potentiostat (PGSTAT 302 N made in Netherlands running NOVA version 1.10.3 software). After the electrodeposition process, the as-electrodeposited α-Co(OH)_2_ on FTO glass substrate was washed several times using Milli-Q water and dried in a vacuum oven at 60 °C overnight. Note, the mass loading of Co(OH)_2_ on FTO substrate can be found in Table S5 of the supplementary information.

### Material characterizations

Both the as-electrodeposited Co(OH)_2_ and the as-disassembled Co(OH)_2_ electrodes were characterized using several techniques to investigate their properties before and after charged/discharged over 2000 cycles. Field-emission scanning electron microscopy (FE-SEM, JSM-7001F (JEOL Ltd., Japan)) with Energy-dispersive X-ray spectroscopy (EDS, X-Max^n^, OXFORD INSTRUMENT, United Kingdom) and transmission electron microscopy (TEM, JEM 1220 (JEOL Ltd., Japan)) were used to study the morphologies of these electrodes. Their crystalline structures were investigated by X-ray diffraction (XRD, Bruker, Germany) using a monochromatic Cu Kα radiation (λ = 0.15405 nm). UV-Visible-Near IR spectrometer (UV/Vis/NIR Lambda 1050, PerkinElmer, U.S.A.), Ultraviolet photoelectron spectrometer (UPS, RIKEN KEIKI, AC-2, U.S.A.), and Fluorescence lifetime spectrometer (FLS, FLS-980, Edinburgh Instruments Ltd., United Kingdom) were used to investigate the optical property. Raman spectrometer (Senterra Dispersive Raman Microscope, Bruker, Germany) with an excitation wavelength of 532 nm and Fourier Transform Infrared Spectrometer (FT-IR, PerkinElmer, U.S.A.) were used to characterize these Co(OH)_2_ electrodes and IL electrolyte. Cyclic voltammetry (CV), Galvanostatic charge discharge (GCD), and electrochemical impedance spectroscopy (EIS) were employed to investigate their electrochemical behaviors under dark and blue LED light illumination (Metrohm AUTOLAB potentiostat, PGSTAT 302 N made in Netherlands running NOVA version 1.10.3 software connecting with blue LED light source 470 nm, light intensity 174 lm at 700 mA).

### Fabrication of a single HECS cell of α-Co(OH)_2_ and the electrochemical evaluation

In this work, the HECS cell of α-Co(OH)_2_ was fabricated as follows. The α-Co(OH)_2_ films on FTO glass substrates were used as both positive and negative electrodes in the HECS cell. The HECS cell was assembled by glue sheet (2 cm × 2 cm) as sealant and a hydroxyl-functionalized polyethylene paper (PE-OH, 1.2 cm × 1.2 cm) as separator. Both the glue sheet and the PE-OH separator were put in the middle of both α-Co(OH)_2_ electrodes. All parts were assembled and then hold with metal clips. After that, the assembled cell was heated at 130 °C for 15 min to melt the glue sheet and then filled 6 M KOH (analytical grad, CARLO ERBA) electrolyte via drilled holes. Finally, the drilled holes were sealed and then further tested with electrochemical technique under dark condition and blue LED illumination. The distance between the blue LED and the HECS cell was fixed at 20 cm for all electrochemical evaluation (CV, GCD, and EIS) and the intensity of blue LED light source was calibrated and fixed at and 700 mA.

### Fabrication of the cell of α-Co(OH)_2_-like DSSC and the current-voltage characterization

To investigate the photoelectric property of α-Co(OH)_2_, the semiconductor/liquid junction photovoltaic cell of α-Co(OH)_2_ was fabricated^5^. The α-Co(OH)_2_ film coated on FTO glass substrates was used as the photoelectrode and the Pt coated on FTO electrode with drilled holes was used as counter electrode. The cell was assembled by sealant sheet (2 cm × 2 cm) by drawing a space area of 1.5 cm × 1.5 cm at the center for the PE-OH separator. The sealant sheet was put in the middle between the α-Co(OH)_2_ electrode and the Pt electrode. All parts were assembled and then hold with metal clips. After that, the assembled cell was heated at 130 °C for 15 min to melt the sealant sheet and then filled the I^−^/I_3_^−^ redox mediator electrolyte via the drilled holes. Finally, the drilled holes were sealed and then tested with current-voltage (I-V) characterization under dark condition and light illumination. The current-voltage curve was obtained by Keithley 2400 source meter (Tektronix, Inc., USA) connected with LabVIEW software. The sunlight simulator was provided by Newport sun simulator 96000 with AM 1.5 G filter. The illumination intensity of 100 mW/cm^2^ was calibrated by BS-520 Si photodiode (Bunnkoukeiki Co., Ltd, Japan).

## Electronic supplementary material

Supplementary Information
