# Supplementary material for: Novel Hybrid Energy Conversion and Storage Cell with Photovoltaic and Supercapacitor Effects in Ionic Liquid Electrolyte
Source: Sci Rep. 2018 Aug 15;8:12192. doi: 10.1038/s41598-018-30707-z (PMC6093932; doi:10.1038/s41598-018-30707-z)
Supplement: Supplementary file 1 — Supplementary Information [file 41598_2018_30707_MOESM1_ESM.pdf]

Supplementary Information

Novel Hybrid Energy Conversion and Storage Cell with Photovoltaic and Supercapacitor Effects in Ionic Liquid Electrolyte

Saran Kalasina[1], Nutthaphon Phattharasupakun[1], Thana Maihom[1], Vinich Promarak[2], Taweesak Sudyoadsuk[2], Jumras Limtrakul[2] and Montree Sawangphruk[1,*]

[1]Department of Chemical and Biomolecular Engineering, School of Energy Science and Engineering, Vidyasirimedhi Institute of Science and Technology, Rayong 21210, Thailand.
[2]Department of Materials Science and Engineering, Vidyasirimedhi Institute of Science and Technology, Rayong 21210, Thailand.

Contents; **Page No.**

Crystallinity of the as-electrodeposited $Co(OH)_2$ S2

Energy-dispersive X-ray spectroscopy (EDS) S3

Raman-FTIR spectra of the [BMpyr][DCA] ionic liquid S4

FLS spectra S5

Electrochemical analysis S6

Calculation details S6

The linear fitting for finding b, k_1, and k_2-values S8

$Co(OH)_2$ electrodes with IL S9

Quantum Chemical Calculations S9

UV-Visible spectra S10

Raman spectra S11

Mechanism of Ni-MH battery S11

Stability test over 4,000 cycles S12

FESEM images S12

CVs and their capacitances S13

The capacitances and Ragone plot S14

The capacities S15

Stability test of the symmetric $Co(OH)_2$ in 2 M KOH electrolyte over 4,000 cycles S15

References S16

1. Crystallinity of the as-electrodeposited $Co(OH)_2$

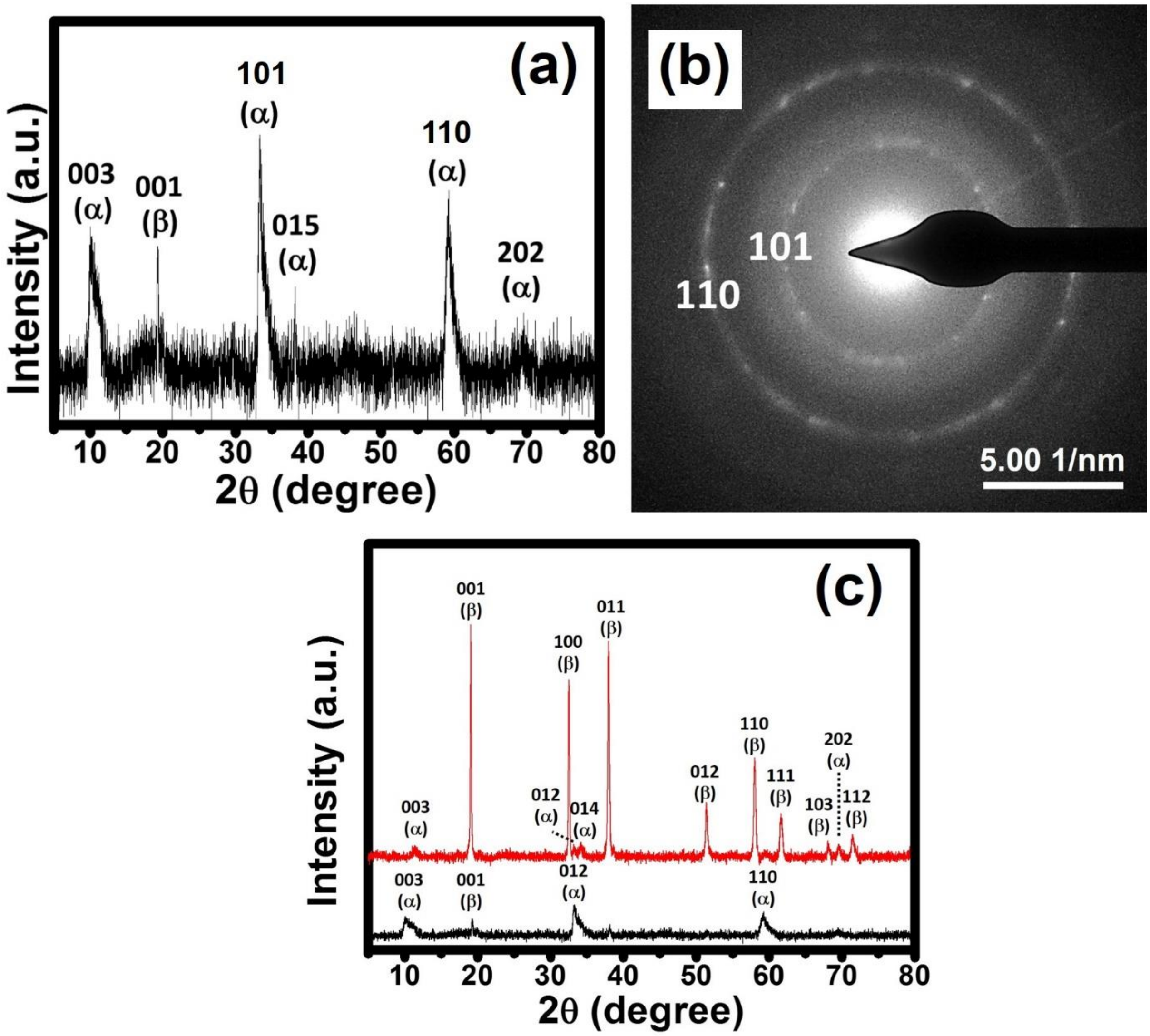

Figure S1. GIXRD pattern (a), the diffraction rings of as-electrodeposited $Co(OH)_2$ (b) and XRD pattern of the powder of $Co(OH)_2$ before (black line) and after (red line) immersed in 6 M KOH (c).

An GIXRD pattern of as-electrodeposited $Co(OH)_2$ film is shown in **Fig.S1a**. The 101 and 110 planes display higher intensity than other peaks. The diffraction ring of the as-electrodeposited $Co(OH)_2$ from TEM technique is shown in **Fig.S1b.** Two diffraction rings can be clearly observed and their SAED analysis can be ascribed to 101 and 110 planes. The XRD patterns of the powder of the as-electrodeposited $Co(OH)_2$ film before (black line) and after (red line) immersed in 6 M KOH are shown in **Figure S1c**. The XRD peaks of α-$Co(OH)_2$ and β-$Co(OH)_2$ are clearly different. The powder of the as-electrodeposited $Co(OH)_2$ film before immersed in 6 M KOH (black line) is mainly α-$Co(OH)_2$, whilst after immersed in 6 M KOH (red line) β-$Co(OH)_2$ is mainly found. From the XRD data, the d-spacing of α-$Co(OH)_2$ calculated from 003 plane ($2\theta = 10.1^{o}$) is *ca.* 8.8 Å. Whist, the d-spacing of β-$Co(OH)_2$ calculated from 001 plane ($2\theta = 19.9^{o}$) is *ca.* 4.5 Å.

2. Energy-dispersive X-ray spectroscopy (EDX)

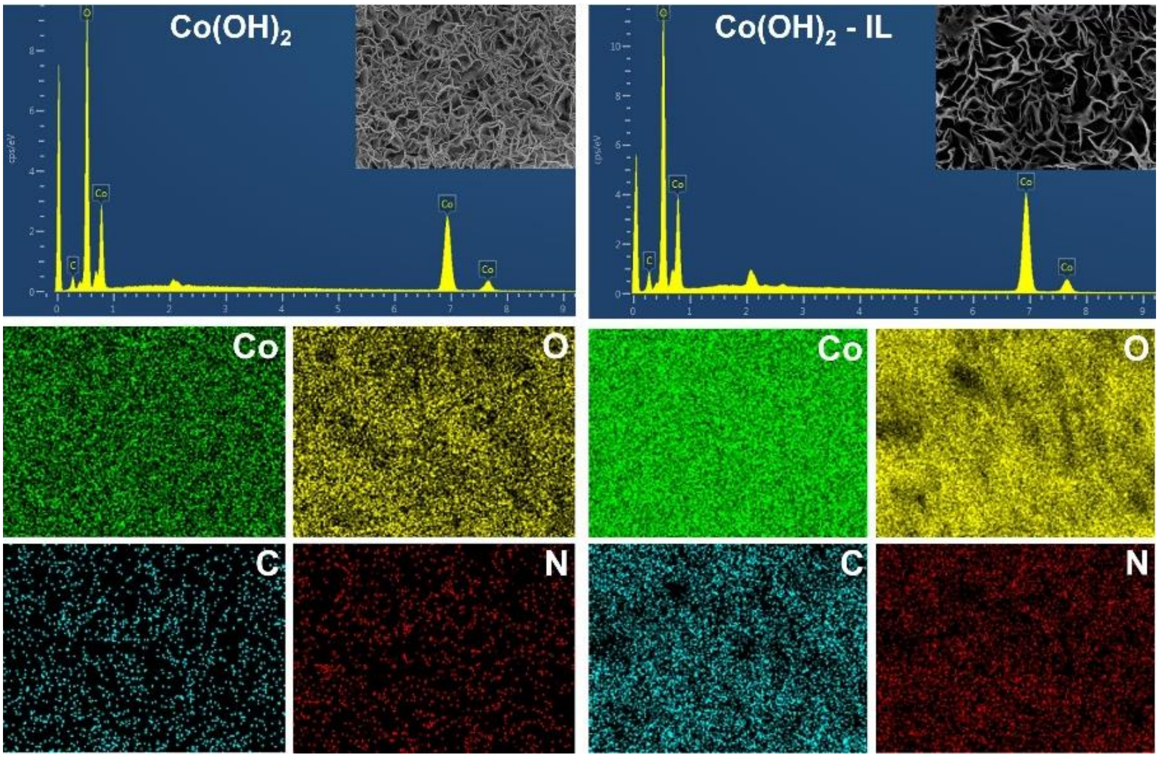

Figure S2. EDX patterns and the elemental mapping analysis (Co, O, C, and N) of the as-electrodeposited $Co(OH)_2$ and the as-tested $Co(OH)_2$ in ionic liquid electrolyte**.**

The $Co(OH)_2$ electrode before and after tested was investigated by EDX technique with the elemental mapping of Co, O, C and N as shown in **Figure S2.** The EDX displays the Co**:**O ratio of 1**:**3**.**

3. Raman-FTIR spectra of the [BMpyr][DCA] ionic liquid

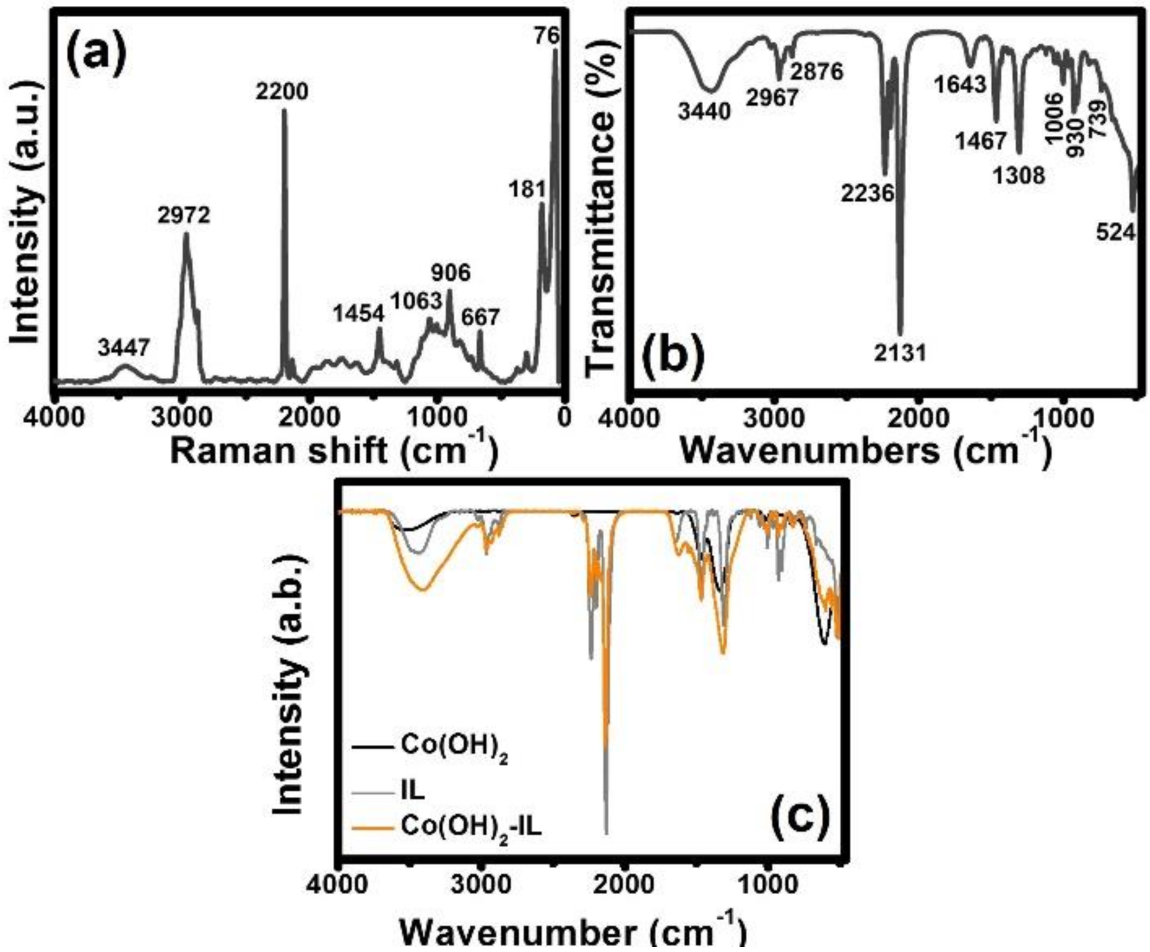

Figure S3. Raman (a), FTIR (b) spectra of the [BMpyr][DCA] IL, and FTIR data of the $Co(OH)_2$ after charged-discharged for 2000 cycles compared with the as-electrodeposited α-$Co(OH)_2$ film and the IL.

To study the effect of ionic liquid on the performance of the symmetric $Co(OH)_2$ supercapacitor, the properties of ionic liquid were investigated with Raman and FTIR techniques. Raman spectrum of the ionic liquid is shown in **Figure S3a.** The peaks at 3447, 2972, and 1063 cm^{-1} can be assigned to O-H, C-H, and C-C stretching, respectively. The peaks at 1454 cm^{-1} can be indexed to CH_2 and CH_3 bending. The strong peak at 2200 cm^{-1} is due to the triple bond between C and N of anion molecules (cyanamide) of IL and the strong peaks at 181 and 76 cm^{-1} can be assigned to the lattice vibration. An FTIR spectrum of the ionic liquid is shown in **Figure S3b.** A broad peak at 3440 cm^{-1} can be assigned to OH^- stretching vibration. The peaks at 2967 and 2876 cm^{-1} can be referred to C-H stretching of CH_2 and CH_3 groups. The strong peaks at 2236 and 2131 cm^{-1} can be indexed to the triple bond between C and N while the peaks at 1643, 1467, and 1308 cm^{-1} relate to the pyrrolidinium cations of IL. Other peaks at around 500-1050 cm^{-1} are due to C-H bending and C-N bonding. To investigate phase deformation of $Co(OH)_2$, the $Co(OH)_2$ film after charged/discharged for 2000 cycles was measured using FTIR technique and compared with the as-prepared α-$Co(OH)_2$ film and IL as shown in **Figure S3c.** The FTIR peaks of the $Co(OH)_2$ film after charged-discharged display the combination between α-$Co(OH)_2$ film and IL.[1] This reflects that IL can maintain the α-phase of $Co(OH)_2$ film after long cycling test.

4. FLS spectra

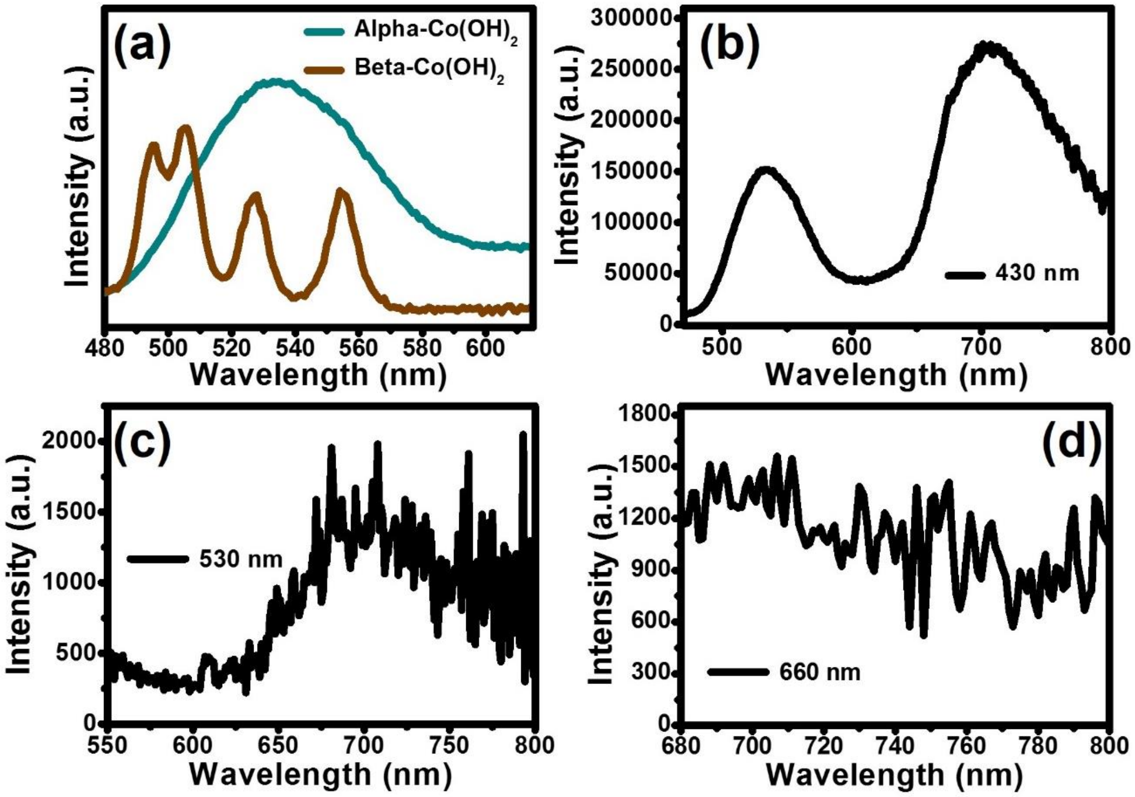

Figure S4. Fluorescence excitation spectra of both α- and β-$Co(OH)_2$ with an excitation wavelength of 430 nm (a), Fluorescence excitation spectra of α-$Co(OH)_2$ with various excitation wavelengths at 430 nm (2.88 eV) (b), 530 nm (2.34 eV) (c), and 660 nm (1.88 eV) (d).

5. Electrochemical analysis

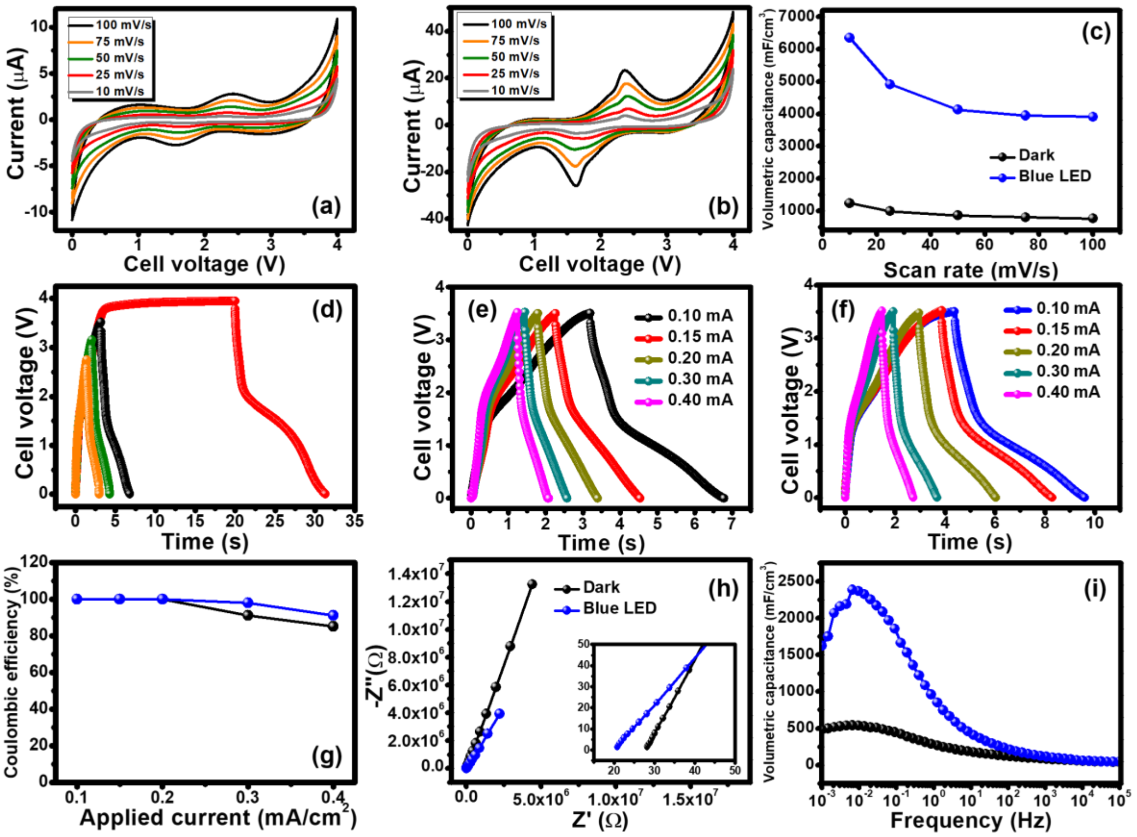

Figure S5. CVs of the $Co(OH)_2$ cell at various scan rates (10, 25, 50, 75, and 100 mV/s) under dark condition (a) and blue LED light illumination (b), the volumetric capacitances as a function of scan rates (c), GCDs at 0.1 mA/cm^2 with various window potentials under dark condition (d), GCDs at various applied currents under dark condition (e) and blue LED light illumination (f), the coulombic efficiency as a function of applied current (g), Nyquist plot (h), and the areal capacitances as a function of frequency (i).

6. Calculation details

6.1 Power law relationship[2, 3, 4, 5];

$$\boldsymbol{i = av^b} \tag{S1}$$

where $\boldsymbol{i}$ is the measured current, $\boldsymbol{v}$ is the scan rate, $\boldsymbol{a}$ and $\boldsymbol{b}$ are adjustable parameters. The b-value can be obtained from the slope of the linear curve between log(i) and log(v).[2]

$$\boldsymbol{i = nFC^{*}D^{\frac{1}{2}}v^{\frac{1}{2}}\left(\frac{\alpha nF}{RT}\right)^{\frac{1}{2}}\pi^{\frac{1}{2}}\chi(bt)} \tag{S2}$$

when $\boldsymbol{i}$ is the current, $\mathbf{C}^*$ is the surface concentration of the electrode material, $\boldsymbol{\alpha}$ is the transfer coefficient, $\boldsymbol{D}$ is the chemical diffusion coefficient, $\boldsymbol{n}$ is the number of electrons involved in the electrode reaction, $\boldsymbol{A}$ is the surface area of the electrode materials, $\boldsymbol{F}$ is the Faraday constant, $\boldsymbol{R}$ is the molar gas constant, $\boldsymbol{T}$ is the temperature, and the function χ(bt) represents the normalized current for a totally irreversible system as indicated by the CV response.

$$\boldsymbol{i = vC_dA} \tag{S3}$$

when $\boldsymbol{C_d}$ is the capacitance, $\boldsymbol{v}$ is the scan rate and $\boldsymbol{A}$ is the surface area of the electrode materials.

$$\boldsymbol{i(V) = k_1 v + k_2 v^{\frac{1}{2}}} \tag{S4}$$

when $\boldsymbol{k_1 v}$ and $\boldsymbol{k_2 v^{\frac{1}{2}}}$ refer to the current contribution from surface capacitive effects and diffusion-controlled intercalation process. $\boldsymbol{k_1}$ and $\boldsymbol{k_2}$ are adjustable parameter at each potential.[6]

6.2 Tuac plot[7];

The optical band gap energy has been determined by using Tauc relation;

$$\boldsymbol{(\alpha h\nu) = k(h\nu - E_g)^n} \tag{S5}$$

where α is the adsorption coefficient, hν is the photon energy, k is a constant value of material, and E_g is the band gap energy. The value of n can be applied with various values such as 1/2 or 2. The absorption spectrum of each $Co(OH)_2$ film was used to estimate the E_g of $Co(OH)_2$. The optical band gap of each $Co(OH)_2$ film was estimated by using linear fitting to find the interception in X-axis.

6.3 Work function or Ionization energy;

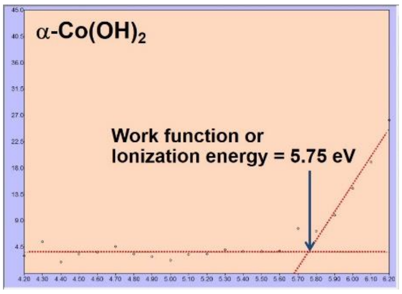

Figure S6. Real curve from RIKEN photoelectron spectroscopy (Model AC-2)

The curve shows the square root of photoelectron emission yield as a function of scan energy. The interception point of the background and the yield line is a photoemission threshold energy, so-called the work function or ionization potential. The work function value correlates with vacuum state (energy level = 0 eV), therefore, this value is normally negative and corresponds to valence band (VB) edge of materials.

7. The linear fitting for finding *b*, k_1, and k_2-values

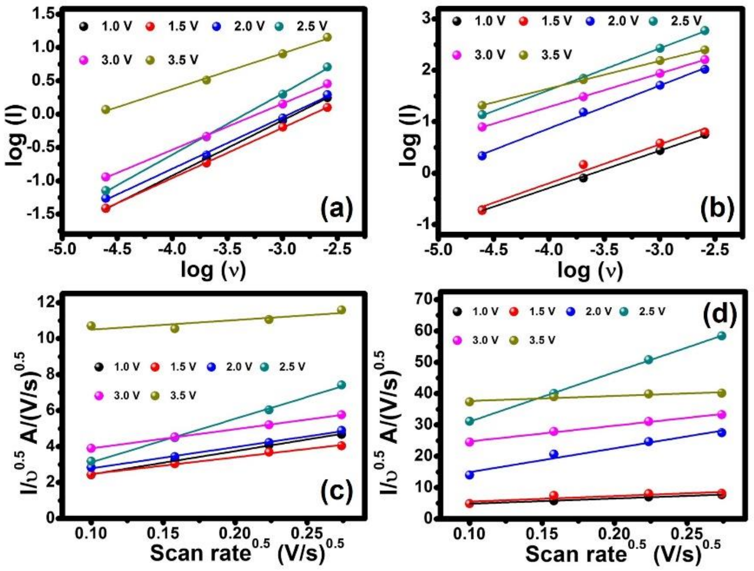

Figure S7. The relationship between log (v) and log (I) under dark condition **(**a) and blue light condition **(**b) and the relationship between the square root of the scan rate and the scan rates under dark condition **(**c) and blue light condition **(**d)**.**

Table S1 Summary of linear fitting for calculating *b*-value.

**Condition**	**Window potential**	**Linear equation**	**R^2**
**Dark**	1.0 V	y = 0.8259x + 2.383	1
(Fig.S6a)	1.5 V	y = 0.7526x + 2.053	0.99
	2.0 V	y = 0.7695x + 2.260	0.99
	2.5 V	y = 0.9169x + 3.061	0.99
	3.0 V	y = 0.6918x + 2.233	0.99
	3.5 V	y = 0.5378x + 2.526	0.99
**Blue**	1.0 V	y = 0.7284x + 2.623	0.99
(Fig.S6b)	1.5 V	y = 0.7561x + 2.827	0.98
	2.0 V	y = 0.8335x + 4.204	0.99
	2.5 V	y = 0.813x + 4.867	0.99
	3.0 V	y = 0.6513x + 3.893	1
	3.5 V	y = 0.537x + 3.794	1

Table S2 Summary of linear fitting for calculating k_1, k_2-values.

**Condition**	**Window potential**	**Linear equation**	**R^2**
**Dark**	1.0 V	y = 12.944x + 1.1587	0.99
(Fig.S6c)	1.5 V	y = 9.2834x + 1.5472	0.99
	2.0 V	y = 11.8685x + 1.599	0.99
	2.5 V	y = 24.229x + 0.702	0.99
	3.0 V	y = 10.673x + 2.832	0.99
	3.5 V	y = 5.3358x + 9.967	0.76
**Blue**	1.0 V	y = 16.627x + 3.193	0.99
(Fig.S6(d))	1.5 V	y = 17.746x + 3.744	0.77
	2.0 V	y = 76.087x + 7.335	0.96
	2.5 V	y = 157.84x + 15.286	1
	3.0 V	y = 50.275x + 19.71	0.99
	3.5 V	y = 16.105x + 36.045	0.91

8. $Co(OH)_2$ electrodes with IL

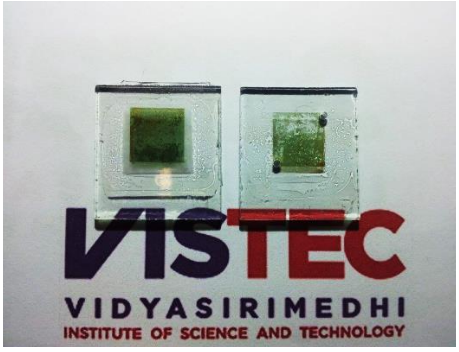

Figure S8. Photographs of the disassembled $Co(OH)_2$ electrodes after charged-discharged for 2000 cycles; photoelectrode side (left) and opposite electrode side (right).

9. Quantum Chemical Calculations

The dicyanamide anion (DCA) molecule, $N(CN)_2^-$, has been theoretically studied by *ab*-initio calculation. The ground-state geometry optimizations were carried out using the MP2 method and the aug-cc-pVTZ basis set. The frequency calculations were also performed at the same level of theory to check the optimized minima of this molecule. All calculations were performed with Gaussian 09 package. The optimized structure of the $N(CN)_2^-$ is shown in **Figure S9**. It has a V-shape molecular configuration with the C1-N1-C2 bond angle of 118.4°. The distances of the both N1-C1 and N1-C2 are equal to 1.32 Å. The highest occupied molecular orbital (HOMO) is found to be delocalized predominantly on all atoms of DCA with π*-orbital (**Figure S10**). The HOMO energy is calculated to be -4.83 eV.

The HOMO energy of other anions in ILs commonly used for battery applications was also calculated by using the same level of theory.[8] The results are shown in **Table S3**. It can be clearly seen that the HOMO energy of the dicyanamide is closest to the valence band of $Co(OH)_2$ (-5.75 eV) as compared to other anions.

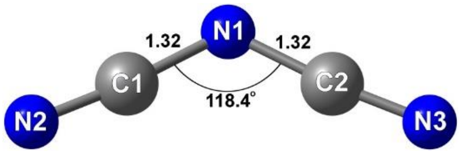

Figure S9. The optimized structure of the dicyanamide anion.

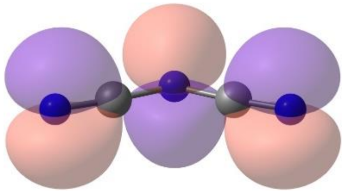

Figure S10. The Highest Occupied Molecular Orbital (HOMO) of the dicyanamide anion.

Table S3. The highest occupied molecular orbital (HOMO) of other anions in ILs calculated with MP2/aug-cc-pvtz level of theory.

Anions in ILs	HOMO (eV)
Dicynayamide (DCA^-)	-4.82
Tricyanomethanide ($C(CN)_3^-$)	-4.63
Chloride (Cl^-)	-4.09
Bromide (Br-)	-3.79
Thiocyanate (SCN^-)	-3.83
Hexafluorophosphate (PF_6^-)	-11.27
Cyanoborate ($B(CN)_4^-$)	-9.04
bis(fluorosulfonyl)imide (FSI^-)	-8.48
bis(tri-fluoromethanesulfonyl)imide ($TFSI^-$)	-8.51

Table S4. The highest occupied molecular orbital (HOMO) of the BMpyr cation in IL calculated with MP2/aug-cc-pvtz level of theory.

Cation in IL	HOMO (eV)
Pyrrolidinium **(**$BMpyr^+$**)**	-16.20

10. UV-Visible spectra

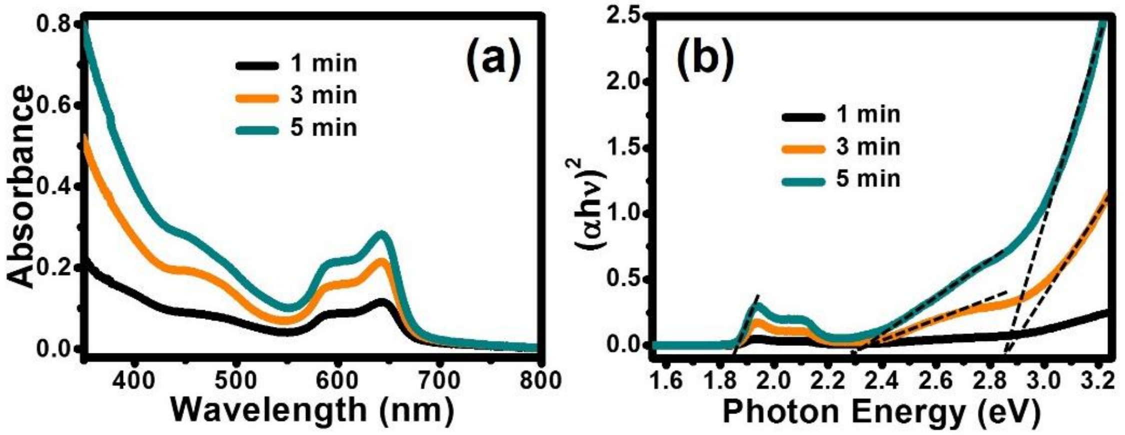

Figure S11. UV-Visible diffuse reflectance spectra of $Co(OH)_2$ films with various electrodeposition times (a) and Tauc plots (b).

11. Raman spectra

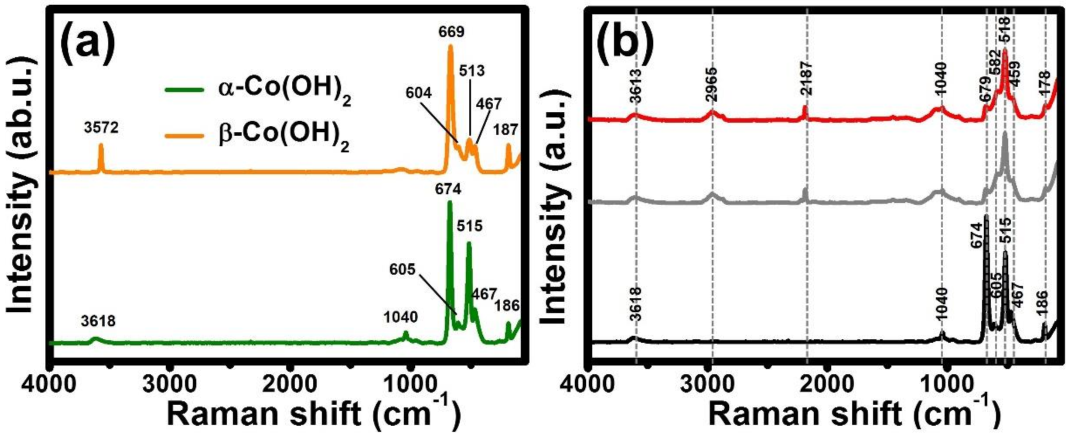

Figure S12. Raman spectra; (a) α-$Co(OH)_2$ (green line) β-$Co(OH)_2$ (brown line) and (b) $Co(OH)_2$ electrode before stability test without IL (black line), $Co(OH)_2$ electrode after stability test with IL (gray line), $Co(OH)_2$ electrode before stability test with IL (red-line).

12. Mechanism of Ni-MH battery

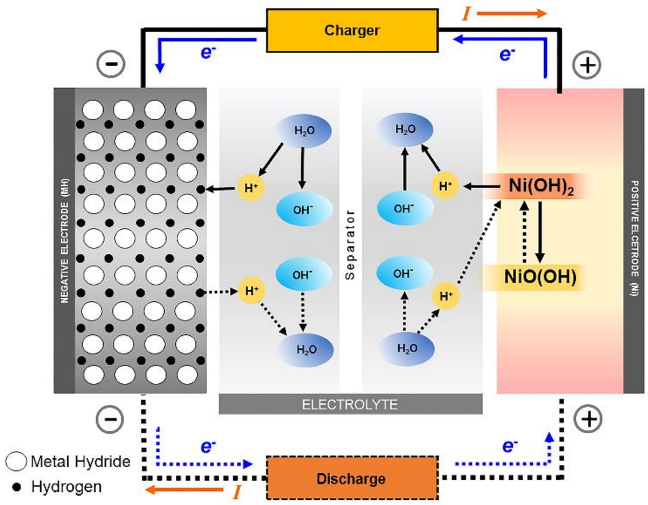

Figure S13. Charge storage mechanism of Ni-MH battery.

13. Stability test over 4,000 cycles

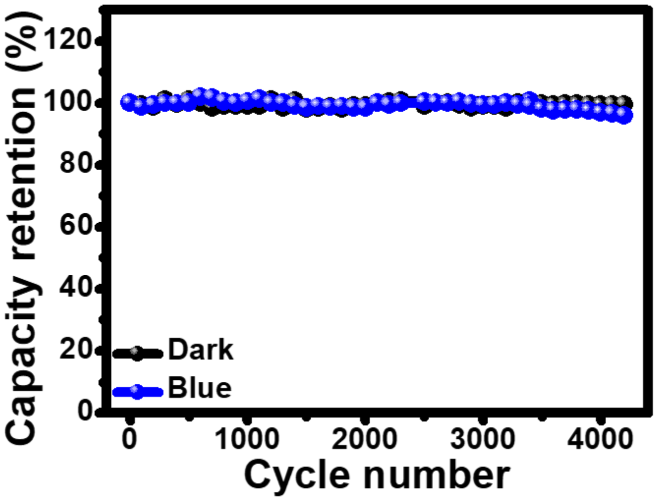

Figure S14. The capacity retention of the symmetric $Co(OH)_2$ HECS in IL electrolyte over 4,000 cycles under dark condition and blue LED light illumination.

14. FESEM images

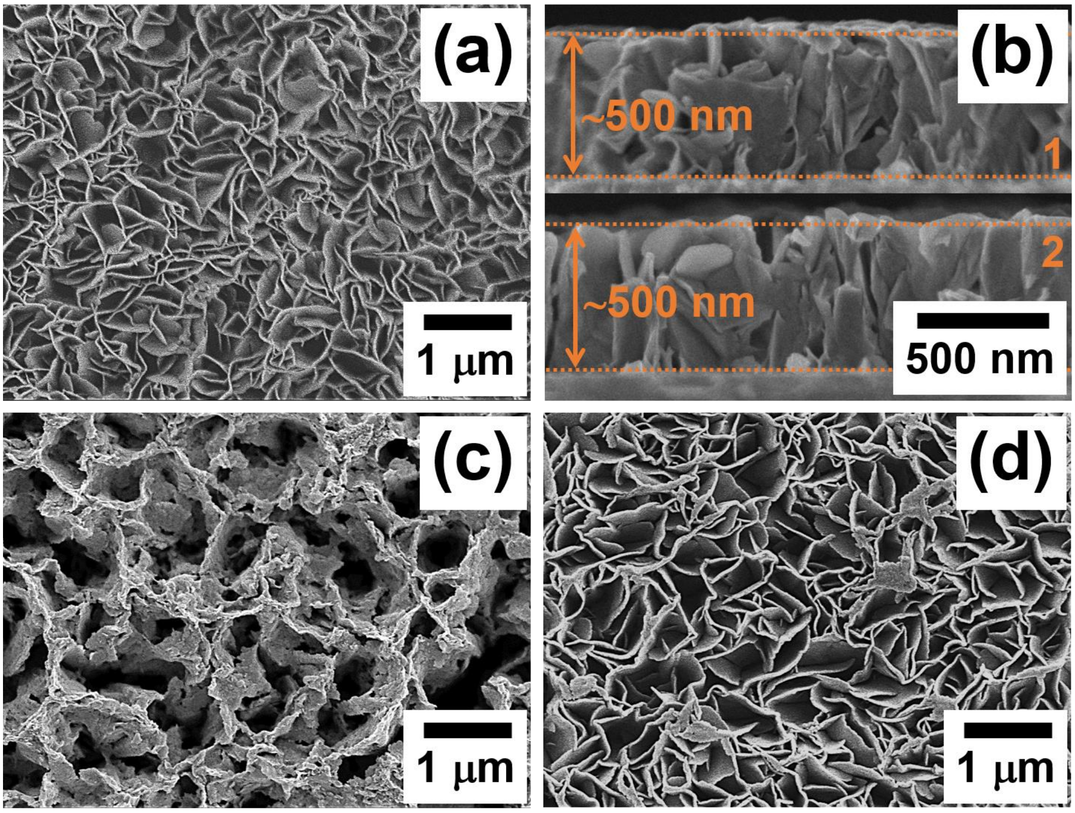

Figure S15. FESEM image of the as-electrodeposited $Co(OH)_2$ film (a), FESEM cross section of the $Co(OH)_2$ films (b), FESEM of the $Co(OH)_2$ film after immersed in 6 M KOH for 5 min (c) and the as-disassembled $Co(OH)_2$ electrode of the ionic liquid-based HECS cell after tested over 2000 cycles (d).

15. CVs and their capacitances

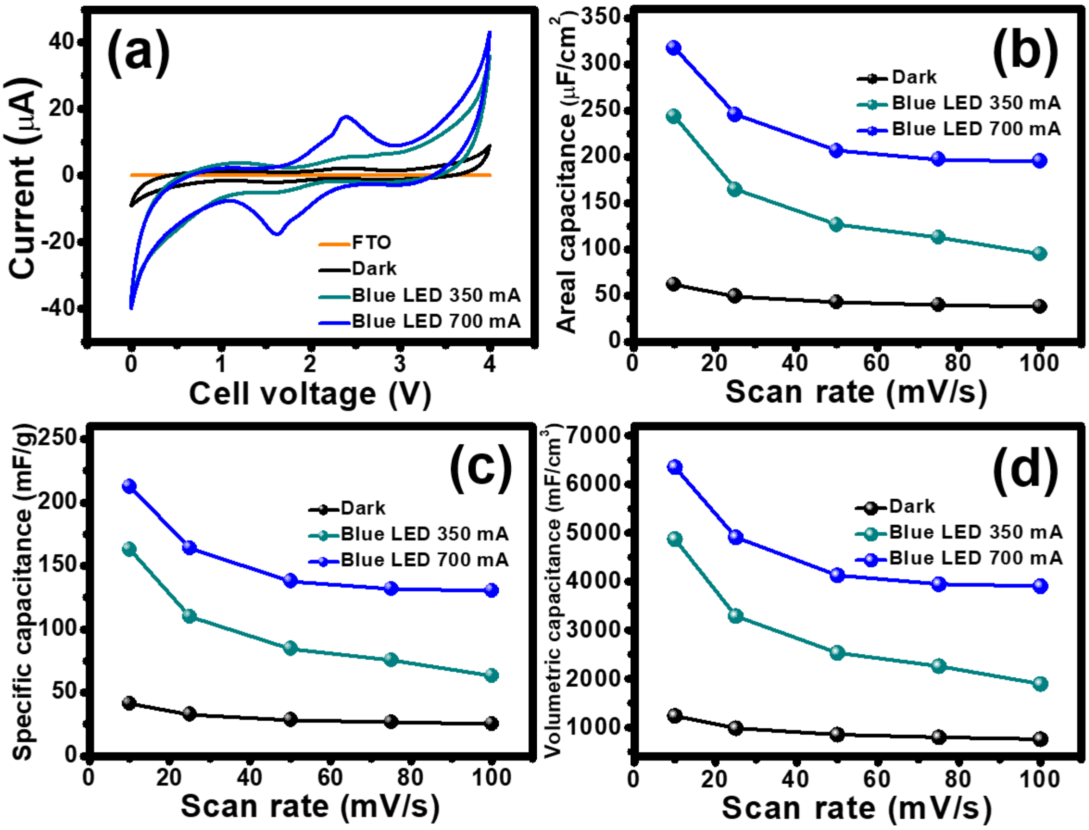

Figure S16. CVs of the symmetric $Co(OH)_2$ HECS in IL electrolyte measured under dark condition (black line) and blue LED light illuminations with different intensities (350 mA in green line and 700 mA in blue line) as compared with FTO glass substrate (a), the areal capacitances as a function of scan rates (b), the specific capacitances as a function of scan rates (c) and the volumetric capacitances as a function of scan rates (d)

16. The capacitances and Ragone plot

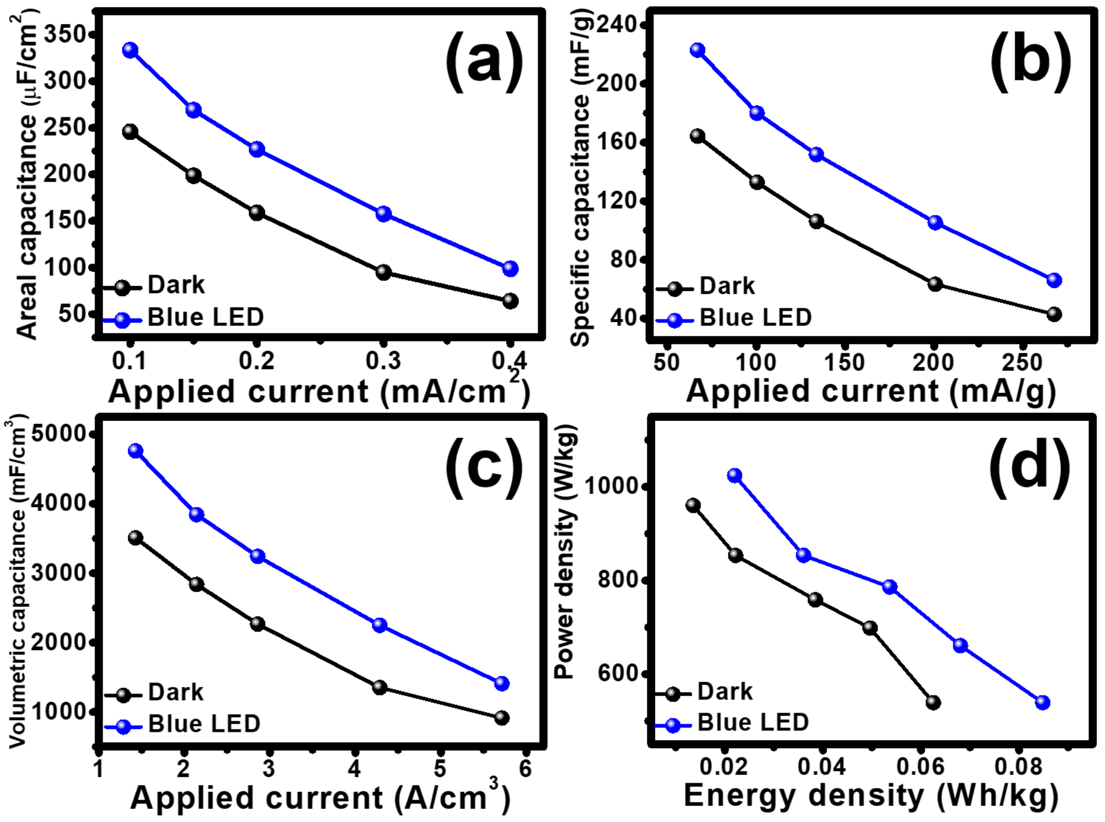

Figure S17. The areal capacitances as a function of the applied currents (a), the specific capacitances as a function of the applied currents (b), the volumetric capacitances as a function of the applied currents (c) and Ragone plot of the symmetric $Co(OH)_2$ HECS in IL electrolyte cell (d).

Table S5 $Co(OH)_2$ mass loading

Electrode	Mass (g)	Testing
1	0.00045	Half-cell
2	0.00047	Half-cell
2	0.00151	Full-cell
3	0.00148	Full-cell

17. The capacities

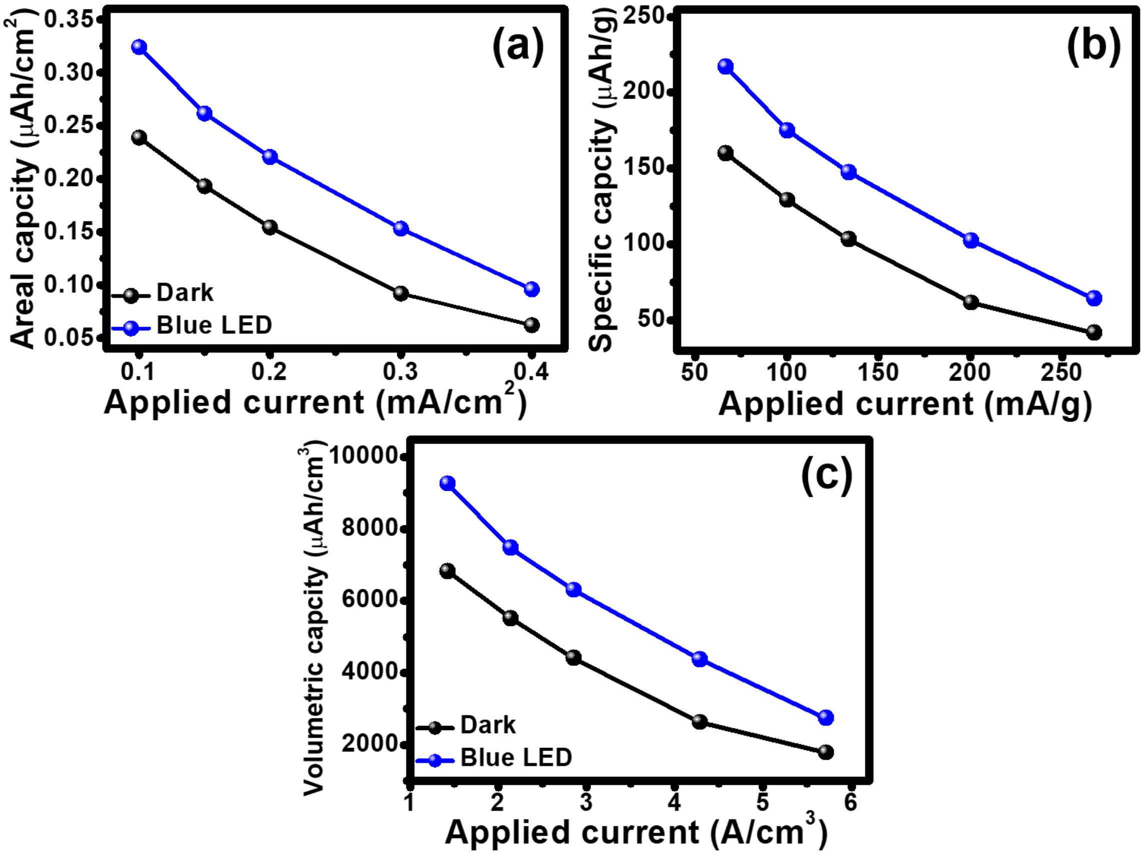

Figure S18. The areal capacities as a function of the applied currents (a), the specific capacities as a function of the applied currents (b), the volumetric capacities as a function of the applied currents (c)

18. Stability test of the symmetric $Co(OH)_2$ in 2 M KOH electrolyte over 4,000 cycles

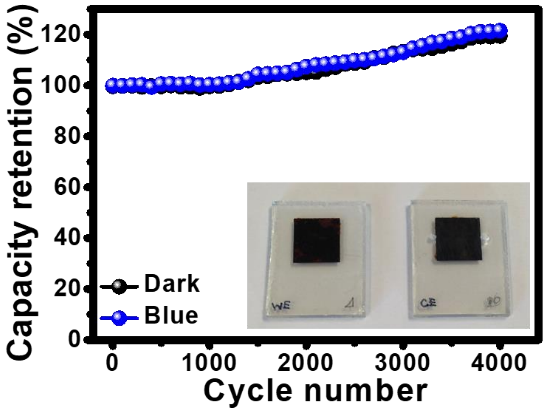

Figure S19. The capacity retention of the symmetric $Co(OH)_2$ in 2 M KOH electrolyte over 4,000 cycles under dark condition and blue LED light illumination. Photographs of the disassembled $Co(OH)_2$ electrodes after charged-discharged for 4000 cycles are shown in the inset; photoelectrode side (left) and opposite electrode side (right).

References

1. Kalasina, S.; Pattanasattayavong, P.; Suksomboon, M.; Phattharasupakun, N.; Wutthiprom, J.; Sawangphruk, M., A new concept of charging supercapacitors based on the photovoltaic effect. *Chem. Commun.* **2017,** *53* (4), 709-712.
2. Lindström, H.; Södergren, S.; Solbrand, A.; Rensmo, H.; Hjelm, J.; Hagfeldt, A.; Lindquist, S.-E., Li+ Ion Insertion in TiO_2 (Anatase). 2. Voltammetry on Nanoporous Films. *J. Phys. Chem. B* **1997,** *101* (39), 7717-7722.
3. Wang, R.; Xu, C.; Lee, J.-M., High performance asymmetric supercapacitors: New NiOOH nanosheet/graphene hydrogels and pure graphene hydrogels. *Nano Energy* **2016,** *19*, 210-221.
4. Augustyn, V.; Simon, P.; Dunn, B., Pseudocapacitive oxide materials for high-rate electrochemical energy storage. *Energy Environ. Sci.* **2014,** *7* (5), 1597-1614.
5. Sathiya, M.; Prakash, A. S.; Ramesha, K.; Tarascon, J. M.; Shukla, A. K., V_2O_5-Anchored Carbon Nanotubes for Enhanced Electrochemical Energy Storage. *J. Am. Chem. Soc.* **2011,** *133* (40), 16291-16299.
6. Wang, J.; Polleux, J.; Lim, J.; Dunn, B., Pseudocapacitive Contributions to Electrochemical Energy Storage in TiO_2 (Anatase) Nanoparticles. *J. Phys. Chem. C* **2007,** *111* (40), 14925-14931.
7. Pinaud, B. A.; Chen, Z.; Abram, D. N.; Jaramillo, T. F., Thin Films of Sodium Birnessite-Type MnO_2: Optical Properties, Electronic Band Structure, and Solar Photoelectrochemistry. *J. Phys. Chem. C* **2011,** *115* (23), 11830-11838.
8. Giffin, G. A., Ionic liquid-based electrolytes for "beyond lithium" battery technologies. *J. Mater. Chem. A* **2016,** *4* (35), 13378-13389.
